# A *Shigella boydii *bacteriophage which resembles *Salmonella *phage ViI

**DOI:** 10.1186/1743-422X-8-242

**Published:** 2011-05-19

**Authors:** Hany Anany, Erika J Lingohr, Andre Villegas, Hans-Wolfgang Ackermann, Yi-Min She, Mansel W Griffiths, Andrew M Kropinski

**Affiliations:** 1 Canadian Research Institute for Food Safety, University of Guelph, ON; N1G 2W1, Canada; 2 Department of Microbiology, Faculty of Science, Ain Shams University, Abbassia, Cairo, Egypt; 3 Laboratory for Foodborne Zoonoses, Public Health Agency of Canada, Guelph, ON; N1G 3W4, Canada; 4 Department of Microbiology, Immunology, and Infectiology, Faculty of Medicine, Laval University, Quebec, QC; G1X 4C6, Canada; 5 Centre for Vaccine Evaluation, Biologics and Genetic Therapies Directorate, Health Canada, Ottawa, ON; K1A 0K9, Canada; 6 Department of Molecular & Cellular Biology, University of Guelph, ON; N1G 2W1, Canada

## Abstract

**Background:**

Lytic bacteriophages have been applied successfully to control the growth of various foodborne pathogens. Sequencing of their genomes is considered as an important preliminary step to ensure their safety prior to food applications.

**Results:**

The lytic bacteriophage, ΦSboM-AG3, targets the important foodborne pathogen, *Shigella*. It is morphologically similar to phage ViI of *Salmonella enterica *serovar Typhi and a series of phages of *Acinetobacter calcoaceticus *and *Rhizobium meliloti*. The complete genome of ΦSboM-AG3 was determined to be 158 kb and was terminally redundant and circularly permuted. Two hundred and sixteen open reading frames (ORFs) were identified and annotated, most of which displayed homology to proteins of *Salmonella *phage ViI. The genome also included four genes specifying tRNAs.

**Conclusions:**

This is the first time that a Vi-specific phage for *Shigella *has been described. There is no evidence for the presence of virulence and lysogeny-associated genes. In conclusion, the genome analysis of ΦSboM-AG3 indicates that this phage can be safely used for biocontrol purposes.

## Background

*Shigella *species *sonnei*, *flexneri*, and *boydii *are among the most important foodborne pathogens [1,2]. Ingestion of food contaminated with these bacteria causes shigellosis within 12 - 48 hours. Fever, aches, fatigue and loss of appetite are the initial symptoms, which may be associated with watery diarrhea that, in turn, may develop into bloody stools or dysentery. A fatal hemolytic-uremic syndrome (HUS), due to the production of Shiga toxin, may also develop in certain severe cases [3]. *Shigella*-related outbreaks occur through direct or indirect human fecal contamination and have been reported in both developed and developing countries wherever poor hygiene standards occur [4]. Food products such as salads, soft cheese, vegetables and meat products are usually reported as being associated with these outbreaks [3].

Lytic phages have been applied successfully to control the growth of various foodborne pathogens including *Shigella *[5]. They are able to attack sensitive bacteria and utilize their host's resources to reproduce. Cell lysis leads to the release of progeny phage particles [6]. As phages are becoming recognized as potential tools to control pathogens in food, phage genomics will play an increasingly important role in ensuring that potentially harmful phage products are selected against [7]. For instance, genomic data could ensure the detection of virulence genes and any genes that might lead to lysogenization of a pathogen [8]. As DNA sequencing techniques advance, the number of sequenced phage genomes has increased exponentially [9]. To date, over 530 complete genome sequences of *Caudovirales *phages have been deposited in the NCBI database, which has allowed detailed comparative analyses with the aim to develop more coherent classification schemes and provide insights into evolutionary processes [10-15].

Several important phages target the Vi or virulence-associated polysaccharide capsular antigen of *Salmonella *Typhi as a receptor [16]. They are classified in seven serotypes (ViI to ViVII). Phage ViII is widely used for phage typing of this bacterium. All Vi phages have isometric heads and either contractile, or long or short noncontractile tails. They belong to the *Myoviridae *(ViI), *Siphoviridae *(ViII), and *Podoviridae *(ViIII to VII), respectively [17]. Phage ViI has a very characteristic morphology that was also found in certain *Acinetobactercalcoaceticus *and *Rhizobium meliloti *viruses [17-20]. Phages of this group possess a neck, a collar, and a contractile tail. They have a thin baseplate, which is connected to a highly ramified structure consisting of either short tail fibers. Phage ViII possesses a lambda-like morphology, but has a base plate with three spikes, while phages ViIII to ViVII have a very short tail terminating in a base plate with at least two spikes [17]. Although Vi phages were first described over 60 years ago, little is known about their molecular structure [16].

In previous work, we isolated three strongly lytic phages that could be considered as good candidates for *Shigella *control (unpublished data). One of them, ΦSboM-AG3, was morphologically similar to phage ViI. We wanted to study some of its growth properties, determine and annotate its genomic sequence, and assess its structural functions in relation to other existing phages in the GenBank. This would aid in a better understanding of the molecular structure of this phage and features that might be useful in its taxonomical classification and application for *Shigella *control.

## Results

### Phage morphology, host range and one-step growth

**Figure 1:**
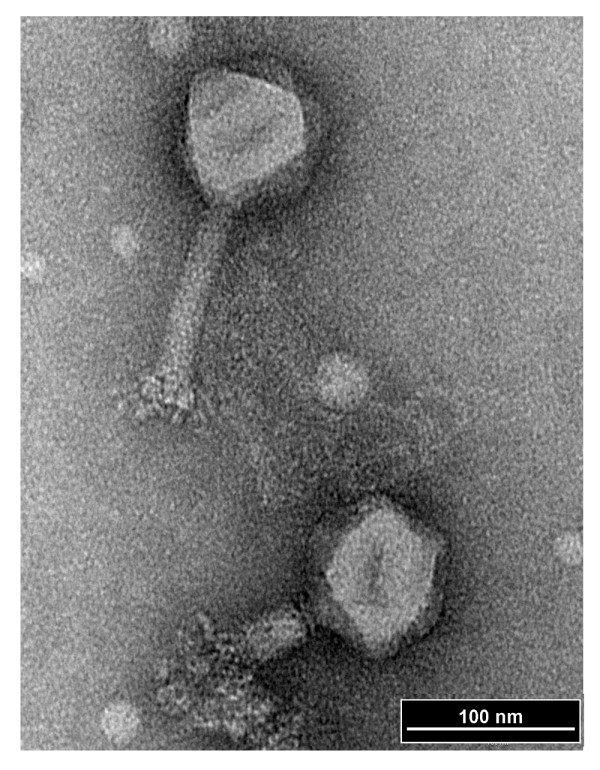
**Electron micrograph of phage ΦSboM-AG3**. The phage has been negatively stained with 2% potassium phosphotungstate. The bar indicates 100 nm.

Testing the collected environmental samples (rinse water, fecal samples, and sewage) against tested *Shigella *strains resulted in the isolation of 24 phages. From these one, ΦSboM-AG3, was chosen for detailed study because of its broad host range and unusual morphology. The negatively stained virus has a clearly defined neck and a collar, and a complex network of baseplate-associated spikes and fibrous structures that is most visible after tail contraction (Figure 1). It has head diameter of approximately 83 nm and a contractile tail 110 nm long by 14 nm in diameter. This indicates that it is a member of the *Myoviridae *[21]. Its morphology is very similar to those of classical phage ViI of *Salmonella enterica *serovar Typhi [17,22] and phage Det7 of *Salmonella enterica *serovar Typhimurium phage [23], but this is the first time that such a phage has been reported to infect *Shigella*.

The host range was determined using a Bioscreen C Plate Reader (Table 1). Most *S. boydii, S. dysenteriae, S. flexneri *and *S. sonnei *strains were infected by ΦSboM-AG3, but the extent of infection varied in each case. The growth of 11 strains out of the 24 tested was totally suppressed in the presence of phage. None of the strains of *E. coli, Salmonella*, or *Listeria *tested were infected by this virus (data not shown).

**Table 1:** Bacterial strains used in this study; and, host range of ΦSboM-AG3 on 24 *Shigella *strains.

*Shigella *strain	Reaction	*Shigella *strain	Reaction
*S. boydii *(C865)	D+	*S. boydii *(79-1109)	C
*S. sonnei *(C866)	C	*S. boydii *(74-3594)	D
*S. flexneri *(C869)	C	*S. boydii *(84-1119)	N
*S. sonnei *(C870)	C	*S. boydii *(83-578)	D+
*S. flexneri *(61-1186)	N	*S. boydii *(99-4528)	D+
*S. flexneri *(71-2747)	C	*S. dysenteriae *(04-3380)	C
*S. flexneri *(04-3435)	C	*S. dysenteriae *(91-3501)	D+
*S. flexneri *(95-3239)	C	*S. dysenteriae *(53-4738)	N
*S. flexneri *(05-3605)	C	*S. dysenteriae *(52-2050)	C
*S. flexneri *(86-3239)	N	*S. dysenteriae *(69-2387)	D+
*S. boydii *(74-1789)	D+	*S. dysenteriae *(94-3065)	C
*S. boydii *(74-4334)	D+	*S. dysenteriae *(79-8006)	D+

C: Complete inhibition of bacterial growth

D+: More than 5 hours delay in bacterial growth to reach log phase compared to control (i.e. Detection time difference ≥5 hours)

D: Less than 5 hours delay in bacterial growth to reach log phase compared to control (i.e. Detection time difference <5 hours)

N: No effect of phage on bacterial growth

**Figure 2:**
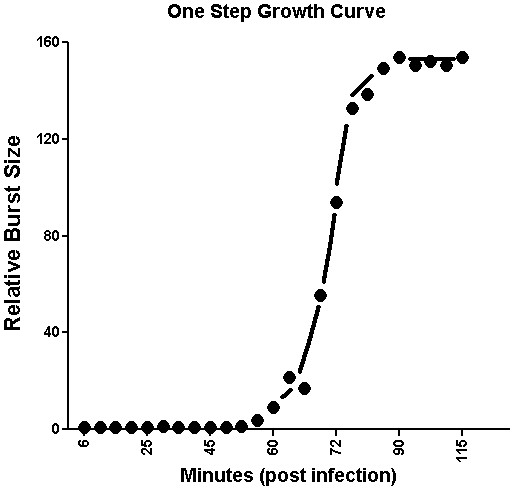
**One-step growth curve of phage ΦSboM-AG3**. *Shigella boydii *C865 was used as the host and the incubation temperature was 30°C

One-step growth studies with ΦSboM-AG3 indicate that at 30°C this phage has a latent period of ~52 min, a rise period of 23 min., and an average burst size of ~152 phages per cell (Figure 2).

### General features of the ΦSboM-AG3 genome

The genome sequence was received as a single contig representing the consensus reads from 19 × coverage. The size of the genome is 158,006 bp with a G+C content of 50.4%; the latter being almost identical to that of its host bacterium, *S. boydii*, (51 mol%). Since pulsed-field gel electrophoresis indicated that the phage genome is approximately 165 kb, the genome of this phage is terminally redundant, with 3.5 kb redundant regions. For comparative purposes with *Salmonella *phage ViI, the ΦSboM-AG3 genome was opened just upstream of the *rIIA *homolog prior to annotation.

### Identification and analysis of open reading frames (ORFs)

The sequence contained no demonstrable frameshifts as indicated by BLASTX analysis. The genome was subjected to automated analysis using AutoFACT complemented analysis for tRNAs; and, by manual annotation using Kodon coupled with BLASTP, PFAM, TMHMM and Phobius analyses of each of its proteins.

**Figure 3:**
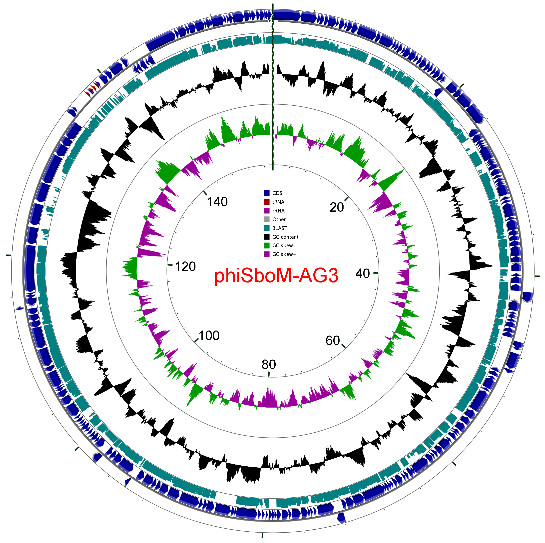
**Genetic and physical map of phage ΦSboM-AG3 as prepared by using CGView **[50]. The outer lane represents genes in the plus strand; tRNA genes are indicated in red. The next lane illustrates genes on the minus strand. The lane with bluish-green boxes represents TBLASTX analysis against the nucleotide sequence of *Salmonella *phage ViI. The lane with black peaks and valleys indicate the GC content, while the innermost lane (green and violet) shows a GC skew analysis.

A total of 216 ORFs were identified in the genome (Figure 3; Additional file 1, Table S1). A total of 146,356 nucleotides (92% of the genome) were involved in coding for putative proteins. Four different start codons were used: ATG, GTG, CTG and TTG at frequencies of 95.4%, 3.2%, 0.9% and 0.5%, respectively. When originally annotated in early 2009, the products of only 107 ORFs possessed homologues with proteins in the nonredundant NCBI database. Detailed comparative proteomic analyses using CoreGenes revealed that this phage shared 46 homologs with *Prochlorococcus *phage P-SSM2 [24], 57 with coliphage T4, 60 with *Aeromonas *phage Aeh1 [25], and, interestingly, 69 with *Delftia acidovorans *phage ΦW-14 [26]. These results suggested that ΦSboM-AG3 was a peripheral member of the T4 superfamily of bacteriophages.

Recent analysis reveals that the majority (179 or 82.8%) of the proteins encoded by this phage show sequence similarity to *Salmonella *phage ViI [22]. The other virus with which ΦSboM-AG3 shares sequence similarity is *Escherichia *coli O157:H7 phage PhaxI, which is represented in GenBank by six fragments (HQ259285 - HQ259290).

### Codon usage and tRNAs

The ΦSboM-AG3 genome was found to contain four tRNA genes for three amino acids, namely, serine (anticodons: TGA & GCT), asparagine (GTT), and tyrosine (GTA). While the manuscript describing the genome of *Salmonella *phage ViI indicates the presence of five tRNAs, these data are not included in their GenBank submission (FQ312032). These two viruses share tRNA-Asn, tRNA-Tyr and tRNA-Ser (GCT), but differ in that ViI has two additions tRNAs for Met (CAT) and Gln (TTG), and ΦSboM-AG3 possesses the second seryl-tRNA. In both viruses, the tRNA genes are to be found in the same relative positions on the genomes. The GC content of the ΦSboM-AG3 tRNAs ranged from 53.9 to 56.2%. Adding these tRNAs to the ORFs resulted in a total coding capacity for the ΦSboM-AG3 genome of 146,481 bp or 92.7%. A comparison of the codon usage pattern of the phage with its host (*Shigella boydii*) showed that none of these tRNAs is likely to enhance translation. We identified six codons which are significantly overrepresented (frequency ≥30% and ≥1.5 fold increase) in phage genes (phenylalanine [UUC], isoleucine [AUG], proline [CCU], lysine [AAG], aspartic acid [GAC] and arginine [CGU]), yet no phage-specified tRNAs exist.

In the following sections we shall briefly discuss the roles of some phage-encoded proteins.

### Nucleotide metabolism, DNA replication and recombination

The ΦSboM-AG3 genome contains numerous genes involved in nucleotide metabolism, DNA replication and recombination. In the former category are a dNTP diphosphatase (*orf063*), a putative nicotinamide phosphoribosyl transferase (*orf149*), NrdA (*orf093*), NrdB (*orf090*) and glutaredoxin (*orf088*) homologs, and thymidylate synthase *orf066*). At least nine genes were identified with play significant roles in DNA replication in coliphage T4 including, a DNA polymerase (*orf236*), a primase (*orf102*), three proteins defined as possessing helicase activity (gp*orf041*/*059*/*125*), and a DNA ligase (*orf045*). Recombination proteins include two topoisomerases (*orf014*/*017*), a T4 gp46/47 recombinase pairs (*orf119*/*121*), and UvsWXY homologs (*orf168*/*061*/*170*).

### Lysis

No holin or lysin-encoding genes were detected in the genome. Holins are usually small proteins characterized by the presence of two or three transmembrane domains. These criteria could apply to products of genes *9.1*, *78*, *117*, *244 *or *246 *which possess 65, 103, 54, 65, and 61 amino acids, respectively. Since holin genes are frequently collocated on phage genomes a detailed PSI-BLAST examination of genes *10, 11, 79, 80, 243*, and *247 *failed to reveal lysin homologs or domains.

### Transcriptional and regulatory sequences

None of the transcriptional regulatory sites were identified in the genome sequence of *Salmonella* phage ViI. Based on sequence homology to the consensus housekeeping *E. coli *promoter recognized by RNA polymerase carrying Sigma 70 (TTGACA (N_15-18_) TATAAT), eight promoters were tentatively identified (Additional file 2, Table S2) which probably function in early transcription.

Initiation of transcription of late genes in T4-related phages involves a complex between core RNA polymerase, a phage encoded sigma factor (gp*55*), an accessory protein (gp*33*) and the sliding clamp protein gp*45 *which "facilitates RNA polymerase recruitment to late promoters" [27].

ΦSboM-AG3 encodes homologs of all three proteins gp*55* (*Orf122*), gp*33 *(*Orf076*) and gp45 (*Orf165*). Based upon the sequence of late promoters (TATAAATA) and allowing one mismatch, 17 putative late promoters were identified in the genome of ΦSboM-AG3. In three cases the putative promoters could result in products which are postulated to be late: P_orf072 _(upstream of T4-like gp*2 *DNA end protector protein), P_orf084 _(gp*5 *baseplate hub subunit), and P_orf217 _(gp*6 *baseplate wedge subunit). A WebLogo [28] was constructed from which the consensus sequence TNT(N3)A(N10)C(N2)ATNAATA was used, with only one mismatch to search the genome for other potential late promoters. Three were identified.

Forty intergenic rho-independent terminators were found, situation that is reminiscent of coliphage T1 (Additional file 2, Table S2) [29].

### DNA packaging and morphogenesis

The morphogenesis of this phage was investigated using genomic (homology searches) and proteomic (SDS-PAGE/mass spectroscopy) approaches. Large and small terminase subunit homologs were determined to be the products of genes *196 *and *198*. Genes for major head, prohead core, portal vertex proteins were identified as genes *185*, *186 *and *191*, respectively. In addition, genes for prohead protease and head completion protein were identified as genes *188 *and *036*, respectively. Genes *037 *and *084 *were for baseplate assembly while, genes *210 *and *212 *showed sequence similarity to tail spike proteins. The tail tube and base plate initiator gene was identified to be gene *073*. Genes *192*, and *195 *are predicted to be genes for tail sheath and tube. Interestingly, the terminase subunits are located between the genes involved in neck, base plate and tailspike synthesis and those involved in head and tail synthesis. The genes associated with baseplate synthesis are located in three widely separated regions of the genome.

**Figure 4:**
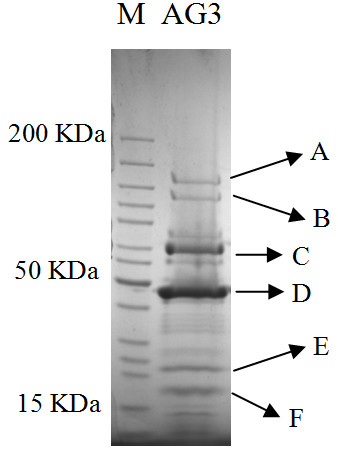
**Denaturing SDS-PAGE gel examination of purified ΦSboM-AG3 particles**. It gives 15 bands of molecular weights ranging from approximately 12 to 126 kDa. Six bands have been analyzed by mass spectrometry (labeled from A to F).

Cesium chloride purified ΦSboM-AG3 virions were analyzed by one-dimensional SDS-PAGE revealing 15 protein bands (Figure 4) with molecular weights of 12.7, 15.6, 17.7, 21.9, 27.2, 32.4, 34.3, 35.7, 37.3, 44.6, 60.4, 67.9, 76.5, 107.5 and 126.3 kDa, based on comparison to size markers. Six of the most intense bands (named A to F) were excised and analyzed by QqTOF mass spectrometry. The observed masses of the protein bands B to F were obviously similar to the predicted sizes by SDS-PAGE (Table 2). The highest sequence coverage was achieved for protein band D (major head protein, 44.6 kDa), which was 64.1%, while the lowest one was 16.4% and was found to be for protein band E (tail protein, 21.9 kDa) due to fewer trypsin cleavage sites. Based on the sequence identification by mass spectrometry, the protein bands C and F were two components (sheath, tube) of tail proteins (products of genes *195*, and *192*, respectively), while the protein band D was assigned to the major head protein (product of gene *185*) (Additional file 3, Table S3). One very interesting observation was the observation that band A with a mass of 126.3 kDa, based upon SDS-PAGE, actually was a proteins of 177.2 kDa. The most obvious explanation is that this protein has a high propensity for reforming a stable tertiary structure which is supported by it being a tailspike protein [30].

**Table 2:** Open reading frames identified by mass spectrometric analysis of CsCl-purified ΦSboM-AG3 phage particles.

SDS-PAGE Band	ORF	Protein description	Molecular weight (kDa)			Number of peptides	% Sequence coverage
			SDS-PAGE	Predicted	MS		
A	206	Conserved hypothetical	126.3	177.2	177.2	36	39.6
B	213	Conserved hypothetical	107.5	106.9	106.9	25	42.6
C	195	Tail sheath (T4 gp*18 *homolog)	67.9	68.2	68.2	18	43.1
D	185	Major capsid (T4 gp*23 *homolog)	44.6	47.9	48.0	23	64.1
E	006	Putative tail protein	21.9	27.6	27.6	6	16.4
F	192	Tail tube (T4 gp*19 *homolog)	17.7	19.8	19.8	12	55.9

## Discussion

ViI-like phages were reported to be found among isolated phages against *Salmonella *Typhi, *Acinetobacter calcoaceticus *and *Rhizobium meliloti *[17,18]. To our knowledge, ΦSboM-AG3 is the first ViI-like phage of *Shigella *species. Although Vi phages were first described almost 80 years ago [17,22], their genome structure has not been investigated until recently. The complete sequences of each member of the Vi-specific *Salmonella *viruses have now been completed. A member of the ViII species, *Salmonella *phage E1 [16], is a siphovirus that has a genome of 45.4 kb. The remainder are all members of the *Autographivirinae *of the *Podoviridae *family [14] with *Salmonella *phage (Vi VI, GenBank accession number FR667955, 38.4 kb), ViIII (39.0 kb), Vi V (38.6 kb), and Vi VII (39.2 kb) belonging to the "T7-like viruses", and ViIV, with a 44.6 kb genome, associated with the "SP6-like viruses" [22].

*Shigella *phage ΦSboM-AG3 possesses three contiguous genes (orfs 207, 210 and 212) which are homologous to tailspike-encoding genes from *Salmonella *phage ViI. This raises the interesting question about what surface receptor is recognized by the *Shigella *phage. The Vi antigen is an acetylated α(1→4) polymer of galactosaminouronic acid units [31,32]. The genes for the biosynthesis of this polymer do not exist in the fully sequenced *S. boydii *strain (NC_010658) as shown by BLAST analyses, nor does the antigen exist on our bacterium (L. Cole, personal communication). Of note was the observation that the serotyping one of the *Shigella *host strains was rough, that is, lipopolysaccharide (LPS) defective. This result suggests that the receptor for ΦSboM-AG3 either lies in the core region of the LPS or is perhaps an outer membrane protein. Phage ViI locus tag Vi01_171c contains a "domain of unknown function" (DUF303) between residues 343-445. This protein motif occurs in proteins described as sialic acid-specific 9-O-acetylesterases and acetylxylan esterases, which suggests that the tail fibers of Vi-specific *Salmonella *phages function to remove acetyl groups from the Vi antigen [22]. The ΦSboM-AG3 homolog, gp210, shows no sequence similarity in this region of the protein, and lacks a DUF303 motif suggesting that its receptor is only peripherally related to the *Salmonella *Vi antigen. The recent paper of Petrov and colleagues [33], re-examining the diversity of T4-like bacteriophages, recognized that ΦSboM-AG3 is a member of the "T4 superfamily". *Salmonella *phage ViI was recognized as being the sole member of a genus by the International Committee on Taxonomy of Viruses (ICTV; Ackermann, personal communication). Since *Shigella *phage ΦSboM-AG3 and *Salmonella *phage ViI are closely related we propose the creation of a new genus, the "ViI-like viruses".

It is notable that the genome of this phage does not contain any genes that are related to bacterial toxins and/or lysogeny. Similar results were obtained when the sequencing of *Listeria *phage P100 DNA revealed that it does not contain any genes or proteins which are known or suspected to be involved in toxicity, pathogenicity or antibiotic resistance [34]. This would make this phage a good candidate for the safe application for controlling foodborne pathogen *Shigella *sp. [8].

## Materials and methods

### Bacteria and bacteriophage

Tryptic Soy Broth (TSB), Tryptic Soy Agar (TSA), and Tryptic Soft Agar (TSB+ 0.4% agar) (Difco Laboratories, Detroit, MI) were used to grow the host bacteria and to propagate the phage. The bacterial strain *Shigella boydii *C865-2 (Canadian Research Institute of Food Safety (CRIFS), University of Guelph, ON, Canada) was used in this study for phage propagation. Other bacterial strains, listed in Table 1, were from the CRIFS collection (strains labeled with the prefix "C") or Nancy Strockbine (Division of Bacterial and Mycotic Diseases, National Center for Infectious Diseases Centers for Disease Control and Prevention, Atlanta, GA, USA).

### Enrichment, isolation and propagation of phages

Sewage samples collected from local waste-water treatment plants (Guelph, ON, Canada) were enriched in an equal volume of TSB and 100 μL of an overnight culture of a mixture of selected strains of *Shigella*. The mixture was incubated for 16-20 h at 30°C with gentle shaking. After incubation, the suspensions were centrifuged at 4000 × g for 15 min at 4°C (Beckman Avanti J-20 XPI, Beckman Coulter Inc., Mississauga, ON, Canada). The supernatant was carefully transferred to another tube and filtered through a sterile disposable filter of 0.45 μm pore size (Fisher Scientific, Mississauga, ON, Canada) and stored at 4°C.

Phages were detected in the enrichments by spot test [35]. Briefly, 100 μL of a bacterial overnight culture was added to 4 mL of molten TSB containing 0.4% agarose at 50-55°C, mixed and poured onto TSA (1.5% agar) plate and allowed to solidify for 15 min. Samples (10 μL) of the filtered were spotting on the top soft agar and allowing it to dry for 20 min before incubation for 16-20 h at 25°C.

After incubation, the plates were examined for the presence of complete or partial lysis zones; these zones were removed from the TSA plates by cutting the soft layer from the plate using a sterile wire loop and placing them separately in 1 mL of λ-Ca^2+ ^phage buffer (λ buffer: 2.5 g/L MgS0_4_. 7H_2_O; 0.05 g/L gelatin; 6 mL/L 1 M Tris buffer; pH 7.2). Following autoclaving at 121°C for 15 min, filter-sterilized CaCl_2_.2H_2_O was added to λ buffer to a final concentration of 5 mM. The tubes were held at room temperature overnight to let the phages diffuse out of the soft agar. The mixture then was filtered through a 0.45 μm membrane filter (Fisher Scientific, Mississauga, ON, Canada) to purify the phages. The isolated phage was purified as previously described [35] by picking up single plaque and using the soft agar overlay method. This procedure was repeated 3 successive times to obtain purified phages.

### Host range determination using a Bioscreen C Plate Reader

The host range of AG3 on 29 *Shigella *strains was determined by measuring the optical density (OD) of the tested bacteria in the presence of phage using the Bioscreen C Microbiology Plate Reader (Labsystems, Helsinki, Finland). The following experimental parameters were used for all experiments: single, wide band (wb) wavelength; 25°C incubation temperature; 5 min preheating time; kinetic measurement; measurement time 24 hours; reading every 20 min and medium intensity shaking for 10 s before measurements. Fifty microliters of the phage lysate were transferred to each of the 100 wells of the sterilized honeycomb plates of the Bioscreen C reader (Growth Curves USA, New Jersey, USA) each of the wells was inoculated with 125 μL of a diluted overnight culture of the tested bacterium (around 10^3 ^CFU/mL). The multiplicity of infection (MOI) was around 100. The control wells contained either phage only, phage buffer only or an equal volume of phage buffer with bacteria. All samples were tested in triplicate. OD data were analyzed using the Bioscreen C data processing software version 5.26 (Labsystems, Helsinki, Finland) to determine the detection time (time required for each test well to increase by 0.3 OD units). Detection times (hr:min) were converted to decimal values, averaged and the mean control detection time was subtracted from all test data for each isolate tested and expressed as detection time difference (DT diff.). Instead of having positive and negative results and based on this time difference, we proposed that the lytic activity of the phages can be classified as; (N): in which phage did not cause any delay in the tested bacterial growth and the growth curve was similar to that of the control; (D): phage cause a delay of tested bacterial growth by less than five hours; DT <5 hrs, (D+): phage cause a delay of the tested bacterial growth by 5 or more hours; DT ≥5 hrs, and (C): in which the phage caused a complete inhibition of bacterial growth after 24 hours.

### One-step growth curve

Burst size and latent period for the selected phages were determined by a one-step growth experiment with some modifications from that described [36].

### Transmission electron microscopy

For electron microscopy, phages were sedimented for 60 min at 25,000 g in a Beckman J2-21 (Palo Alto, Ca) centrifuge using a JA-18.1 fixed angle rotor. This was followed by two washings in 0.1 M neutral ammonium acetate under the same conditions. Purified phages were deposited on carbon-coated Formvar films on copper grids, stained with 2% potassium phosphotungstate (pH 7.0), and examined in a Philips EM 300 electron microscope operated at 60 kV. Magnification was monitored with T4 phage tails.

### DNA isolation and sequencing

A crude phage lysate was freed from bacterial debris by centrifugation at 14,000 × g for 20 min at 4°C. Contaminating nucleic acids in the supernatant were digested with pancreatic DNase 1, and RNase A, each to a final concentration of 10 μg/mL (Sigma-Aldrich Canada Ltd., Oakville, ON) and phage particles were precipitated in the presence 10% w/v (final concentration) PEG-8000 and 1 M NaCl at 4°C overnight. The precipitated phage particles were recovered by centrifugation, resuspended in TM buffer (10 mM Tris-HCl, pH 7.8, 1 mM MgSO_4_) and purified by separation on a self-generating cesium chloride (CsCl) gradient (1.5 g/ml CsCl, run at 21,000 × g at 4°C for 24 h) in a fixed angle, Beckman SW 90Ti rotor. Following purification by a second passage through a CsCl gradient for another 24 hours, the phage was dialyzed against two changes of two liters each of 1 × 10 mM TE buffer (pH 8.0), using Pierce dialysis cassettes with 3500 molecular weight cut-off (Thermo Scientific, Fisher Scientific, Mississauga, ON), and stored at 4°C. The DNA was extracted from a portion of the purified viral particles using the SDS/proteinase K method modified from [35] followed by extraction with phenol:chloroform:isoamyl alcohol (25:24:1, V/V), ethanol precipitation and resolution in 10 mM Tris-HCl (pH 7.5). The DNA was characterized spectrophotometrically.

The DNA was subjected to pyrosequencing (454 technology) at the McGill University and Genome Québec Innovation Centre (Montreal, QC, Canada) to a coverage of 19×.

### Genome annotation

Prior to annotation, the genome was opened immediately upstream of the *rIIA *gene so that it could be compared with the sequence of the related ViI phage. The genome was initially subjected to automated annotation using AutoFACT [37], following which all open reading frames (ORFs) were confirmed using Kodon version 2.0 (Applied Maths Inc., Austin, TX, USA). Genes were identified from among the predicted coding sequences based on the presence of ATG, GTG, CTG or TTG start codons, followed by at least 30 additional codons, and an upstream sequence resembling the following ribosome-binding site, GGAGGT [38,39]. Phage-encoded tRNA genes were identified with tRNAScan-SE and Aragorn, using the default parameters [40,41].

Batch PFAM motif searches [42] were made at http://pfam.sanger.ac.uk/search#searchBatchBlock, while determination of the protein molecular weight and isoelectric point employed http://greengene.uml.edu/programs/FindMW.html.

The BLASTP algorithm was used to determine the similarity to described proteins in the National Center for Biotechnology Information [NCBI] database with searches conducted using Batch BLAST (http://greengene.uml.edu/programs/NCBI_Blast.html). DNAMAN was used to determine the codon usage information of both phage ΦSboM-AG3 and its host *S. boydii*. Promoters were identified based on sequence similarity to the consensus *E. coli *promoter, TTGACA (N_15-18_) TATAAT, immediately upstream of an annotated gene [43]. Rho-independent terminators were discovered by examining the secondary structure of the DNA adjacent to polyT sequences using MFOLD [44]. In addition we employed WebGeSTer [45] at http://pallab.serc.iisc.ernet.in/gester/rungester.html. Only terminators with a ΔG of less than -10 kcal/mol are reported. Genomic comparisons at the proteomic level were made using CoreGenes [46,47]. Transmembrane domains were predicted using TMHMM v2.0 and Phobius [48,49].

### Proteomic analyses

The intact phage particles were lysed using Laemmli's sample buffer (4% SDS, 20% glycerol, 10% 2-mercaptoethanol, 0.004% bromophenol blue, 0.125 M Tris HCl) and boiled for 5 min [35]. The solubilized proteins were subsequently separated by a 12.5% SDS-polyacrylamide gel electrophoresis, and stained with SimplyBlue SafeStain (Invitrogen Canada, Burlington, ON). The gel data were analyzed using BioNumerics software (Applied Maths). The six most intense phage bands were excised and subjected to mass spectrometric analysis at the Mass Spectrometry Facility of Queen's University (Kingston, ON, Canada).

### Genome sequence

The annotated genome sequence for the *Shigella *phage ΦSboM-AG3 was deposited in the NCBI nucleotide database under the accession number FJ373894.

## Abbreviations

gp: gene product, used in the context of coliphage T4 proteins e.g. gp*18*, gp*19*, and gp*23 *are the protein products of genes *18*, *19 *and 23, respectively; MALDI: matrix-assisted laser desorption ionization; QqTOF MS: quadrupole time-of-flight mass spectrometry; MS/MS: tandem mass spectrometry.

## Competing interests

The authors declare that they have no competing interests.

## Authors' contributions

HA, MG, HWA and AMK contributed to the writing of this manuscript. AV carried out the AutoFACT analyses; HA and AMK finalized the annotation; YM carried out the proteomic analyses assisted by EJL; and the electron microscopy was carried out in collaboration with HWA. MG was the principal investigator and provides all facilitates to complete this work. All the authors read and approved the final manuscript.

## Authors' information

HA is currently a post-doctoral fellow in Canadian Research Institute for Food Safety, Food Science Department, University of Guelph, Ontario, Canada.

## Supplementary Material

Additional file 1 **Table S1**. **Characteristics of the ΦSboM-AG3 genes and their products**. BLASTP and PFAM searches conducted on January 12, 2011. The minimum E value used for reporting the PFAM values was 8.4 e-05. TMD = transmembrane domains. Click here for file

Additional file 2 **Table S2**. **Putative promoter and rho-independent terminators found in the ΦSboM-AG3 genome**. Click here for file

Additional file 3 **Table S3. Details of mass spectrometric analysis of ΦSboM-AG3 proteins**. Click here for file
