# Supplementary material for: A Shigella boydii bacteriophage which resembles Salmonella phage ViI
Source: Virol J. 2011 May 19;8:242. doi: 10.1186/1743-422X-8-242 (PMC3121705; doi:10.1186/1743-422X-8-242)
Supplement: Additional file 1 — Table S1. Characteristics of the ΦSboM-AG3 genes and their products. BLASTP and PFAM searches conducted on January 12, 2011. The minimum E value used for reporting the PFAM values was 8.4 e-05. TMD = transmembrane domains. [file 1743-422X-8-242-S1.DOC]

Additional Table S1. Characteristics of the φSboM_AG3 genes and their products.

| **Gene** | **Coordinates** | **Length** | **Strand** | **Mass** | **pI** | **AA Length** | **Product** | **Homology & Motifs** |
| --- | --- | --- | --- | --- | --- | --- | --- | --- |
| orf001 | 1 - 2757 | 2757 | + | 105807 | 6.1 | 918 | RIIA protein | CBW37869.1| RIIA protector from prophage-induced early lysis [Salmonella phageVi01] |
| orf002 | 2789 - 4351 | 1563 | + | 57675 | 5.8 | 520 | RIIB protein | CBW37870.1| RIIB Protector from prophage-induced early lysis [Salmonella phage Vi01] |
| orf003 | 4403 - 4708 | 306 | + | 11973 | 6.9 | 101 | conserved hypothetical protein | CBW37871.1| putative uncharacterised protein [Salmonella phage Vi01] |
| orf004 | 4677 - 5102 | 426 | + | 15297 | 4.9 | 141 | conserved hypothetical protein | CBW37872.1| putative uncharacterised protein [Salmonella phage Vi01] |
| orf005 | 5132 - 5524 | 393 | + | 14968 | 5.4 | 130 | conserved hypothetical protein | CBW37873.1| unnamed protein product [Salmonella phage Vi01] & BAH15177.1| hypothetical protein [Serratia phage KSP90] |
| orf006 | 5503 - 6312 | 810 | + | 27619 | 4.3 | 269 | putative tail protein | CBW37874.1| putative tail fibre [Salmonella phage Vi01]; PF00047.18 ig Domain & PF02368.11 Big_2 Family |
| orf008 | 6315 - 6545 | 231 | + | 8698 | 5.1 | 76 | conserved hypothetical protein | CBW37875.1| putative uncharacterised protein [Salmonella phage Vi01] |
| orf009 | 6634 - 7146 | 513 | + | 19277 | 8.7 | 170 | putative histone-like protein | CBW37876.1| putative uncharacterised protein [Salmonella phage Vi01] |
| orf009.1 | 7196 - 7393 | 198 | + | 7299 | 9.7 | 65 | conserved hypothetical membrane protein | CBW37877.1| putative uncharacterised protein [Salmonella phage Vi01]; two TMD |
| orf010 | 7436 - 7924 | 489 | + | 19014 | 9.1 | 162 | conserved hypothetical protein | CBW37878.1| putative uncharacterised protein [Salmonella phage Vi01] |
| orf011 | 7921 - 8502 | 582 | + | 21662 | 5.4 | 193 | conserved hypothetical protein | CBW37879.1| putative uncharacterised protein [Salmonella phage Vi01] |
| orf014 | 8552 - 10465 | 1914 | + | 71823 | 7.2 | 637 | topoisomerase II, large subunit | CBW37880.1| DNA topoisomerase 2 [Salmonella phage Vi01]; PF02518.19 HATPase_c & PF00204.18 DNA_gyraseB Domains |
| orf015 | 10467 - 10913 | 447 | + | 16630 | 4.8 | 148 | hypothetical protein |  |
| orf017 | 10906 - 12246 | 1341 | + | 51185 | 5.6 | 446 | gp52 topoisomerase II, medium subunit | ADQ55849.1| topoisomerase II medium subunit [Escherichia phage PhaxI] & CBW37881.1| Topoisomerase IIM [Salmonella phage Vi01]; PF00521.13 DNA_topoisoIV Family |
| orf018 | 12289 - 12576 | 288 | + | 10815 | 4.1 | 95 | conserved hypothetical membrane protein | ADQ55850.1| hypothetical protein gp024 [Escherichia phage PhaxI] & CBW37882.1| putative uncharacterised protein [Salmonella phage Vi01]; one TMD |
| orf020 | 12579 - 12890 | 312 | + | 11596 | 4.7 | 103 | conserved hypothetical protein | ADQ55851.1| hypothetical protein gp025 [Escherichia phage PhaxI] |
| orf021 | 12894 - 13304 | 411 | + | 15391 | 7.9 | 136 | Arn.3 conserved hypothetical protein | ADQ55852.1| hypothetical protein gp026 [Escherichia phage PhaxI] & CBW37883.1| conserved uncharacterised protein [Salmonella phage Vi01] |
| orf022 | 13355 - 13597 | 243 | + | 9286 | 6.7 | 80 | conserved hypothetical protein | CBW37885.1| putative uncharacterised protein [Salmonella phage Vi01] & ADQ55854.1| hypothetical protein gp028 [Escherichia phage PhaxI] |
| orf024 | 13594 - 14193 | 600 | + | 21827 | 4.3 | 199 | Tk.4 protein | ADQ55855.1| hypothetical protein gp029 [Escherichia phage PhaxI] & CBW37886.1| conserved uncharacterised protein [Salmonella phage Vi01] |
| orf026 | 14194 - 14826 | 633 | + | 24373 | 5.4 | 210 | DexA exonuclease | CBW37888.1| DexA exonuclease A? [Salmonella phage Vi01] |
| orf027 | 14823 - 15128 | 306 | + | 11518 | 7.8 | 101 | conserved hypothetical membrane protein | CBW37889.1| putative uncharacterised protein [Salmonella phage Vi01]; one TMD |
| orf028 | 15193 - 15534 | 342 | + | 13147 | 6.7 | 113 | conserved hypothetical protein | CBW37892.1| putative uncharacterised protein [Salmonella phage Vi01] |
| orf029 | 15534 - 16094 | 561 | + | 21643 | 6.4 | 186 | putative serine/threonine protein phosphatase | CBW37894.1| putative uncharacterised protein [Salmonella phage Vi01] |
| orf030 | 16129 - 16542 | 414 | + | 15507 | 8.3 | 137 | conserved hypothetical protein | YP_003387109.1| hypothetical protein Slin_2289 [Spirosoma linguale DSM 74] |
| orf031 | 16539 - 16877 | 339 | + | 12661 | 9.3 | 112 | conserved hypothetical protein | CBW37896.1| putative uncharacterised protein [Salmonella phage Vi01] |
| orf032 | 16948 - 17262 | 315 | + | 12242 | 5.1 | 104 | conserved hypothetical protein | CBW37897.1| putative uncharacterised protein [Salmonella phage Vi01] |
| orf033 | 17262 - 17768 | 507 | + | 18520 | 6.6 | 168 | Cd, allosteric enzyme activated by HM-dCTP and dCTP for dUMP synthesis | CBW37898.1| conserved uncharacterised protein [Salmonella phage Vi01]; PF00383.15 dCMP_cyt_deam_1 Family |
| orf034 | 17779 - 18186 | 408 | + | 15188 | 9.7 | 135 | conserved hypothetical membrane protein | CBW37899.1| conserved uncharacterised protein [Salmonella phage Vi01]; PF03703.7 DUF304 Domain; one TMD |
| orf035 | 18189 - 18401 | 213 | + | 8104 | 6.8 | 70 | conserved hypothetical protein | CBW37900.1| conserved uncharacterised protein [Salmonella phage Vi01] |
| orf036 | 18398 - 19012 | 615 | - | 24258 | 9.5 | 204 | gp4, head completion protein | CBW37901.1| Gp4 head completion protein [Salmonella phage Vi01] |
| orf037 | 19063 - 20031 | 969 | + | 36144 | 8.4 | 322 | gp48, putative tail tube associated base plate protein | CBW37902.1| Gp48 T4-like baseplate tail tube cap [Salmonella phage Vi01] |
| orf038 | 20043 - 20597 | 555 | + | 21519 | 4.6 | 184 | gp53, baseplate wedge subunit | CBW37903.1| Gp53 baseplate wedge subunit [Salmonella phage Vi01] & ADQ55839.1| baseplate wedge subunit [Escherichia phage PhaxI]; ; PF11246.1 Phage_gp53 Family |
| orf039 | 20594 - 21982 | 1389 | + | 52730 | 5.1 | 462 | conserved hypothetical protein | ADQ55838.1| hypothetical protein gp014 [Escherichia phage PhaxI] & CBW37904.1| conserved phage associated protein [Salmonella phage Vi01] |
| orf040 | 21994 - 23940 | 1947 | + | 70684 | 5.1 | 648 | conserved hypothetical protein | CBW37905.1| phage associated protein [Salmonella phage Vi01] & ADQ55837.1| hypothetical protein gp013 [Escherichia phage PhaxI] |
| orf041 | 23941 - 24606 | 666 | - | 26463 | 6.9 | 221 | gp59, T4-like loader of gp41 DNA helicase | ADQ55836.1| loader of gp41 DNA helicase [Escherichia phage PhaxI] & CBW37906.1| Gp59 Loader of T4-like helicase [Salmonella phage Vi01]; PF08993.3 T4-helicase_N Domain |
| orf042 | 24599 - 25294 | 696 | - | 27138 | 10.0 | 231 | putative homing endonuclease | YP_002922087.1| Homing endonuclease [Enterobacteria phage JSE] |
| orf043 | 25291 - 25629 | 339 | - | 13329 | 10.0 | 112 | conserved hypothetical protein | CBW37908.1| putative uncharacterised protein [Salmonella phage Vi01] & ADQ55834.1| hypothetical protein gp011 [Escherichia phage PhaxI] |
| orf044.1 | 25595 - 25852 | 258 | - | 9681 | 8.8 | 85 | hypothetical membrane protein | one TMD |
| orf044 | 25854 - 26087 | 234 | - | 9032 | 9.8 | 77 | hypothetical membrane protein | one TMD |
| orf045 | 26056 - 27522 | 1467 | - | 54853 | 5.2 | 488 | gp30 DNA ligase | CBW37911.1| Gp30 DNA ligase [Salmonella phage Vi01]; PF01068.14 DNA_ligase_A_M Domain |
| orf046 | 27578 - 27730 | 153 | - | 5792 | 11.0 | 50 | hypothetical membrane protein | one TMD |
| orf047.1 | 27727 - 27894 | 168 | - | 5851 | 11.1 | 55 | hypothetical membrane protein | one TMD |
| orf047 | 27891 - 28076 | 186 | - | 7433 | 10.6 | 61 | hypothetical membrane protein | one TMD |
| orf048 | 28076 - 28618 | 543 | - | 21017 | 7.0 | 180 | conserved hypothetical membrane protein | CBW37913.1| putative uncharacterised protein [Salmonella phage Vi01]; one TMD |
| orf049 | 28661 - 29269 | 609 | - | 22137 | 6.0 | 202 | e.6 conserved hypothetical protein | CBW37914.1| conserved uncharacterized protein [Salmonella phage Vi01]; PF04012.5 PspA_IM30 Family |
| orf050 | 29285 - 29626 | 342 | - | 12389 | 6.9 | 113 | conserved hypothetical protein | CBW37915.1| putative uncharacterised protein [Salmonella phage Vi01] & ADQ55833.1| hypothetical protein gp010 [Escherichia phage PhaxI] |
| orf051 | 29681 - 30031 | 351 | - | 13409 | 7.7 | 116 | conserved hypothetical protein | CBW37916.1| putative uncharacterised protein [Salmonella phage Vi01] & ADQ55831.1| hypothetical protein gp08 [Escherichia phage PhaxI] |
| orf053 | 30033 - 30245 | 213 | - | 7415 | 8.3 | 70 | conserved hypothetical protein | CBW37917.1| putative uncharacterised protein [Salmonella phage Vi01] & ADQ55830.1| hypothetical protein gp07 [Escherichia phage PhaxI] |
| orf054 | 30245 - 30361 | 117 | - | 4252 | 10.3 | 38 | conserved hypothetical membrane protein | CBW37918.1| putative uncharacterised protein [Salmonella phage Vi01]; one TMD |
| orf055 | 30358 - 31551 | 1194 | - | 46591 | 6.7 | 397 | conserved hypothetical protein | CBW37919.1| conserved uncharacterised protein [Salmonella phage Vi01] & ADQ55827.1| hypothetical protein gp04 [Escherichia phage PhaxI] |
| orf057 | 31679 - 31999 | 321 | - | 11918 | 4.9 | 106 | conserved hypothetical protein | ADQ55826.1| hypothetical protein gp03 [Escherichia phage PhaxI] & CBW37920.1| putative uncharacterised protein [Salmonella phage Vi01] |
| orf058 | 32015 - 32350 | 336 | - | 12874 | 5.3 | 111 | conserved hypothetical protein | CBW37921.1| putative uncharacterised protein [Salmonella phage Vi01] & ADQ55825.1| hypothetical protein gp02 [Escherichia phage PhaxI] |
| orf059 | 32413 - 33840 | 1428 | - | 53979 | 5.3 | 475 | gp41, DNA primase-helicase subunit | ADQ55824.1| DNA primase-helicase subunit [Escherichia phage PhaxI] & CBW37922.1| Gp41 DNA primase-helicase subunit [Salmonella phage Vi01]; PF03796.8 DnaB_C Domain |
| orf060 | 33847 - 34176 | 330 | - | 12059 | 6.6 | 109 | conserved hypothetical protein | ADQ55823.1| hypothetical protein gp01 [Escherichia phage PhaxI] & CBW37923.1| putative uncharacterised protein [Salmonella phage Vi01] |
| orf061 | 34154 - 35239 | 1086 | - | 40741 | 5.1 | 361 | UvsX RecA-like recombination protein | CBW37924.1| UvsX RecA-like recombination protein [Salmonella phage Vi01]; PF00154.14 RecA Family |
| orf062 | 35224 - 35766 | 543 | - | 20168 | 5.0 | 180 | conserved hypothetical protein | CBW37925.1| putative uncharacterised protein [Salmonella phage Vi01] |
| orf063 | 35766 - 36320 | 555 | - | 21153 | 5.0 | 184 | putative dUTP diphosphatase | CBW37926.1| 2-Deoxyuridine 5-triphosphate nucleotidohydrolase (dUTPase [Salmonella phage Vi01]; PF08761.4 dUTPase_2 Domain |
| orf065 | 36317 - 36886 | 570 | - | 22281 | 5.4 | 189 | hypothetical protein | CBW37927.1| putative uncharacterised protein [Salmonella phage Vi01] |
| orf066 | 36883 - 37929 | 1047 | - | 39154 | 6.1 | 348 | putative thymidylate synthase | CBW37928.1| Thymidylate synthase [Salmonella phage Vi01]; PF00303.12 Thymidylat_synt Domain |
| orf067 | 37929 - 38591 | 663 | - | 24526 | 9.1 | 220 | conserved hypothetical protein | CBW37929.1| putative uncharacterised protein [Salmonella phage Vi01] |
| orf069 | 38666 - 39532 | 867 | - | 30473 | 4.8 | 288 | conserved hypothetical protein | CBW37930.1| putative uncharacterised protein [Salmonella phage Vi01] |
| orf070 | 39709 - 39996 | 288 | - | 11156 | 4.6 | 95 | conserved hypothetical protein | CBW37931.1| putative uncharacterised protein [Salmonella phage Vi01] |
| orf071 | 40017 - 40766 | 750 | - | 28807 | 5.2 | 249 | conserved hypothetical protein | CBW37932.1| putative uncharacterised protein [Salmonella phage Vi01] |
| orf072 | 40829 - 41533 | 705 | - | 28432 | 10.0 | 234 | gp2 DNA end protector protein | CBW37933.1| Gp2 DNA end protector protein [Salmonella phage Vi01] |
| orf073 | 41587 - 42531 | 945 | + | 35138 | 4.9 | 314 | gp54 baseplate tail tube initiator | CBW37934.1| Gp54-baseplate tail tube [Salmonella phage Vi01]; PF06841.5 Phage_T4_gp19 Family |
| orf074 | 42558 - 43589 | 1032 | - | 39125 | 5.2 | 343 | gp32 T4-like ssDNA binding protein | CBW37935.1| Gp32 single-strand DNA binding protein [Salmonella phage Vi01]; PF08804.3 gp32 Domain |
| orf075 | 43688 - 43927 | 240 | - | 9035 | 5.5 | 79 | conserved hypothetical protein | CBW37936.1| putative uncharacterised protein [Salmonella phage Vi01] |
| orf076 | 43935 - 44180 | 246 | - | 8979 | 5.1 | 81 | gp33 T4-like late promoter transcription accessory protein | CBW37937.1| Gp33 late promoter transcription factor [Salmonella phage Vi01] |
| orf077 | 44173 - 44298 | 126 | - | 4154 | 10.0 | 41 | conserved hypothetical protein | CBW37938.1| Regulatory protein, FmdB family [Salmonella phage Vi01] |
| orf078 | 44405 - 44716 | 312 | - | 12028 | 9.5 | 103 | conserved hypothetical membrane protein | CBW37939.1| putative uncharacterised protein [Salmonella phage Vi01]; two TMD |
| orf079 | 44716 - 45306 | 591 | - | 22797 | 5.4 | 196 | conserved hypothetical protein | CBW37940.1| putative uncharacterised protein [Salmonella phage Vi01]; PF02075.10 RuvC Domain |
| orf080 | 45358 - 45849 | 492 | - | 17946 | 4.8 | 163 | hypothetical protein | CBW37941.1| putative uncharacterised protein [Salmonella phage Vi01] |
| orf081 | 45827 - 46351 | 525 | - | 20387 | 6.2 | 174 | hypothetical protein | CBW37942.1| putative uncharacterised protein [Salmonella phage Vi01] |
| orf082 | 46551 - 47207 | 657 | + | 24861 | 4.9 | 218 | gp26 baseplate hub subunit | CBW37943.1| Gp26 base plate hub subunit [Salmonella phage Vi01]; PF12322.1 T4_baseplate Family |
| orf083 | 47207 - 47356 | 150 | + | 5865 | 5.1 | 49 | putative protein | no identifiable ribosome-binding site |
| orf084 | 47718 - 49328 | 1611 | + | 58181 | 5.3 | 536 | gp5 baseplate hub subunit and tail lysozyme | CBW37944.1| Gp5-tail associated lysozyme [Salmonella phage Vi01]; PF05257.9 CHAP & PF06714.4 Gp5_OB Domains |
| orf085 | 49401 - 49781 | 381 | + | 14041 | 4.8 | 126 | putative gp25, baseplate wedge subunit | CBW37945.1| Gp25 base plate wedge protein [Salmonella phage Vi01]; PF04965.7 GPW_gp25 Domain |
| orf086 | 49778 - 50104 | 327 | - | 12236 | 8.8 | 108 | hypothetical protein | CBW37946.1| putative uncharacterised protein [Salmonella phage Vi01] |
| orf087 | 50134 - 50595 | 462 | - | 17632 | 5.4 | 153 | hypothetical protein | CBW37947.1| putative uncharacterised protein [Salmonella phage Vi01] |
| orf088 | 50701 - 50925 | 225 | - | 8275 | 8.9 | 74 | putatitve glutaredoxin | CBW37949.1| Glutaredoxin [Salmonella phage Vi01]; PF00462.17 Glutaredoxin Domain |
| orf090 | 50935 - 52038 | 1104 | - | 42358 | 5.3 | 367 | NrdB, ribonucleotide reductase subunit beta | CBW37950.1| Ribonucleotide-diphosphate reductase beta subunit [Salmonella phage Vi01]; PF00268.14 Ribonuc_red_sm Domain |
| orf091 | 52040 - 52333 | 294 | - | 10935 | 6.9 | 97 | hypothetical protein | CBW37951.1| putative uncharacterised protein [Salmonella phage Vi01] |
| orf093 | 52404 - 54683 | 2280 | - | 85966 | 5.6 | 759 | NrdA, ribonucleoside-diphosphate reductase, alpha subunit | CBW37952.1| Ribonucleoside-diphosphate reductase 1 subunit alpha [Salmonella phage Vi01]; PF02867.8 Ribonuc_red_lgC Family & PF00317.14 Ribonuc_red_lgN & PF03477.9 ATP-cone Domains |
| orf095 | 54769 - 55611 | 843 | - | 31437 | 5.6 | 280 | PhoH-like protein | CBW37953.1| PhoH-like phosphate starvation-inducible protein [Salmonella phage Vi01]; PF02562.9 PhoH Family |
| orf096 | 55663 - 56355 | 693 | - | 27062 | 9.8 | 230 | putative homing endonuclease | YP_002922087.1| Homing endonuclease [Enterobacteria phage JSE]; PF01844.16 HNH Family |
| orf097 | 56442 - 57236 | 795 | - | 28650 | 8.9 | 264 | conserved hypothetical protein | CBW37954.1| phage-encoded peptidoglycan binding protein [Salmonella phage Vi01]; PF11860.1 DUF3380 Family & PF01471.11 PG_binding_1 Domain |
| orf098 | 57306 - 57605 | 300 | - | 11020 | 4.8 | 99 | hypothetical protein |  |
| orf099 | 57602 - 57790 | 189 | - | 7018 | 4.9 | 62 | conserved hypothetical protein | CBW37956.1| conserved uncharacterized protein [Salmonella phage Vi01] |
| orf100 | 57845 - 58333 | 489 | - | 17215 | 9.6 | 162 | hypothetical protein | CBW37957.1| putative uncharacterised protein [Salmonella phage Vi01] |
| orf101 | 58529 - 58735 | 207 | - | 7677 | 5.0 | 68 | hypothetical protein |  |
| orf102 | 58732 - 59790 | 1059 | - | 40936 | 9.1 | 352 | gp61 DNA primase subunit | CBW37958.1| Gp61 DNA primase subunit [Salmonella phage Vi01] |
| orf103 | 59790 - 60401 | 612 | - | 23431 | 8.9 | 203 | conserved hypothetical protein | CBW37959.1| conserved uncharacterised protein [Salmonella phage Vi01] |
| orf104 | 60459 - 60812 | 354 | - | 13194 | 5.4 | 117 | MobD.6 conserved hypothetical protein | CBW37960.1| conserved phage associated protein [Salmonella phage Vi01] |
| orf105 | 60863 - 61102 | 240 | - | 8863 | 9.3 | 79 | hypothetical protein |  |
| orf107 | 61112 - 61681 | 570 | - | 20277 | 6.3 | 189 | conserved hypothetical protein | CBW37961.1| phage associated protein [Salmonella phage Vi01] |
| orf109 | 61739 - 63460 | 1722 | - | 63990 | 7.7 | 573 | conserved hypothetical protein | CBW37962.1| phage associated protein [Salmonella phage Vi01] |
| orf110.1 | 63481 - 63621 | 141 | - | 5438 | 12.0 | 46 | conserved hypothetical protein | CBW37962.1| phage associated protein [Salmonella phage Vi01] |
| orf110 | 63627 - 64118 | 492 | - | 18388 | 9.4 | 163 | hypothetical protein |  |
| orf111 | 64194 - 64529 | 336 | - | 12797 | 4.2 | 111 | hypothetical protein |  |
| orf112 | 64552 - 64827 | 276 | - | 10642 | 6.1 | 91 | conserved hypothetical protein | CBW37963.1| putative uncharacterised protein [Salmonella phage Vi01] & ADQ55848.1| hypothetical protein gp023 [Escherichia phage PhaxI] |
| orf113 | 64935 - 65573 | 639 | - | 22882 | 4.3 | 212 | conserved hypothetical protein | ADQ55847.1| hypothetical protein gp022 [Escherichia phage PhaxI] & CBW37964.1| phage associated protein [Salmonella phage Vi01] |
| orf114 | 65573 - 65962 | 390 | - | 14661 | 10.0 | 129 | Vs.1 conserved hypothetical protein | CBW37965.1| conserved uncharacterised protein [Salmonella phage Vi01] & ADQ55846.1| hypothetical protein gp021 [Escherichia phage PhaxI]; PF10715.2 REGB_T4 Family |
| orf114.1 | 66087 - 66221 | 135 | - | 5310 | 6.5 | 44 | hypothetical protein |  |
| orf115 | 66287 - 66565 | 279 | - | 10427 | 4.7 | 92 | conserved hypothetical protein | ADQ55845.1| hypothetical protein gp020 [Escherichia phage PhaxI] & CBW37966.1| putative uncharacterised protein [Salmonella phage Vi01] |
| orf116 | 66546 - 66875 | 330 | - | 12923 | 8.7 | 109 | NrdA.1 conserved hypothetical protein | CBW37967.1| NrdA.1 [Salmonella phage Vi01] |
| orf117 | 66872 - 67036 | 165 | - | 6308 | 8.7 | 54 | hypothetical membrane protein | two TMD |
| orf119 | 67039 - 69375 | 2337 | - | 88400 | 5.1 | 778 | gp46 recombination endonuclease subunit | CBW37969.1| Gp46 recombination endonuclease subunit [Salmonella phage Vi01]; PF02463.12 SMC_N Family |
| orf121 | 69377 - 70492 | 1116 | - | 42928 | 5.0 | 371 | gp47 recombination protein subunit | CBW37970.1| Gp47 recombination protein subunit [Salmonella phage Vi01]] |
| orf122 | 70479 - 71270 | 792 | - | 30608 | 5.5 | 263 | gp55, T4-like sigma factor involved in late transcription | CBW37971.1| Gp55 sigma factor for late transcription [Salmonella phage Vi01] |
| orf123 | 71282 - 71722 | 441 | - | 16662 | 5.9 | 146 | putative Ribonuclease HI | CBW37972.1| ribonuclease H [Salmonella phage Vi01]; PF00075.17 RnaseH Domain |
| orf124 | 71855 - 72622 | 768 | + | 28172 | 4.6 | 255 | conserved hypothetical protein | CBW37973.1| conserved phage associated protein [Salmonella phage Vi01] |
| orf125 | 72619 - 74337 | 1719 | - | 64977 | 6.2 | 572 | putative ATP-dependent helicase | CBW37974.1| ATP-dependent helicase [Salmonella phage Vi01]; PF04851.8 ResIII Family |
| orf127 | 74466 - 74744 | 279 | - | 9724 | 9.5 | 92 | putative DNA-binding protein | CBW37975.1| DNA binding protein [Salmonella phage Vi01]; PF00216.14 Bac_DNA_binding Domain |
| orf128 | 74838 - 75086 | 249 | - | 9235 | 6.5 | 82 | hypothetical protein |  |
| orf129 | 75083 - 75361 | 279 | - | 10240 | 4.8 | 92 | conserved hypothetical protein | CBW37980.1| phage associated protein [Salmonella phage Vi01] |
| orf130 | 75363 - 76046 | 684 | - | 26094 | 5.9 | 227 | conserved hypothetical protein | CBW37981.1| putative uncharacterised protein [Salmonella phage Vi01] |
| orf131 | 76104 - 77009 | 906 | - | 33816 | 9.3 | 301 | conserved hypothetical protein | CBW37982.1| putative uncharacterised protein [Salmonella phage Vi01] |
| orf132 | 77061 - 77375 | 315 | - | 11674 | 4.8 | 104 | conserved hypothetical protein | CBW37983.1| putative uncharacterised protein [Salmonella phage Vi01] |
| orf133 | 77375 - 78061 | 687 | - | 25470 | 8.3 | 228 | conserved hypothetical protein | CBW37984.1| putative uncharacterised protein [Salmonella phage Vi01] |
| orf134 | 78141 - 78590 | 450 | - | 17271 | 9.8 | 149 | conserved hypothetical protein | CBW37985.1| phage assiociated protein [Salmonella phage Vi01] |
| orf135 | 78624 - 78782 | 159 | - | 6517 | 10.1 | 52 | hypothetical membrane protein | one TMD |
| orf136 | 78784 - 79071 | 288 | - | 10711 | 4.3 | 95 | hypothetical protein |  |
| orf137 | 79068 - 79442 | 375 | - | 13361 | 9.0 | 124 | hypothetical protein |  |
| orf138 | 79502 - 79741 | 240 | - | 9005 | 9.9 | 79 | conserved hypothetical protein | CBW37988.1| conserved uncharacterised protein [Salmonella phage Vi01 |
| orf139 | 79838 - 80122 | 285 | - | 11086 | 4.1 | 94 | hypothetical protein |  |
| orf141 | 80224 - 80550 | 327 | - | 11881 | 4.2 | 108 | conserved hypothetical protein | CBW37990.1| acyl carrier protein [Salmonella phage Vi01] |
| orf144 | 80593 - 82794 | 2202 | - | 80984 | 5.0 | 733 | conserved hypothetical protein | CBW37991.1| putative uncharacterised protein [Salmonella phage Vi01] |
| orf146 | 82787 - 82957 | 171 | - | 6436 | 4.2 | 56 | hypothetical protein |  |
| orf147 | 82959 - 83318 | 360 | - | 13418 | 4.8 | 119 | hypothetical protein | YP_004009936.1| hypothetical protein CC31p078 [Enterobacteria phage CC31] |
| orf149 | 83361 - 85004 | 1644 | - | 60775 | 5.5 | 547 | putative nicotinamide phosphoribosyl transferase | YP_001193099.1| putative nicotinate phosphoribosyltransferase [Flavobacterium johnsoniae UW101] & YP_024539.1| putative nicotinamide phosphoribosyl transferase [Staphylococcus phage K]; PF04095.9 NAPRTase Family |
| orf152 | 85115 - 85909 | 795 | - | 30060 | 6.3 | 264 | conserved hypothetical protein | CBW37992.1| conserved uncharacterised protein [Salmonella phage Vi01] & ADQ55843.1| hypothetical protein gp018 [Escherichia phage PhaxI] |
| orf153 | 85890 - 86291 | 402 | - | 15089 | 9.3 | 133 | conserved hypothetical protein | CBW37994.1| conserved uncharacterised protein [Salmonella phage Vi01]; PF11672.1 DUF3268 Family |
| orf155 | 86352 - 86723 | 372 | - | 14114 | 5.6 | 123 | conserved hypothetical protein | CBW37995.1| conserved uncharacterised protein [Salmonella phage Vi01] |
| orf158 | 86768 - 88786 | 2019 | - | 75661 | 5.9 | 672 | conserved hypothetical protein | CBW37997.1| conserved uncharacterised protein [Salmonella phage Vi01] |
| orf160 | 88902 - 90122 | 1221 | - | 47068 | 7.9 | 406 | conserved hypothetical protein | CBW37998.1| conserved uncharacterised protein [Salmonella phage Vi01] |
| orf161 | 90192 - 91061 | 870 | - | 32308 | 4.6 | 289 | conserved hypothetical protein | CBW37999.1| conserved uncharacterised protein [Salmonella phage Vi01] |
| orf162 | 91078 - 91542 | 465 | - | 18044 | 8.8 | 154 | RegA translational repressor protein | CBW38000.1| putative uncharacterised protein [Salmonella phage Vi01] & ADO99628.1| translational repressor [Prochlorococcus phage Syn33]; PF01818.10 Translat_reg Domain |
| orf163 | 91572 - 91994 | 423 | - | 16158 | 9.6 | 140 | gp62 clamp loader subunit, DNA polymerase accessory protein | CBW38001.1| clamp holder for DNA polymerase [Salmonella phage Vi01] |
| orf164 | 91999 - 92988 | 990 | - | 37410 | 5.7 | 329 | gp44 clamp loader subunit, DNA polymerase accessory protein | CBW38002.1| Gp44-sliding clamp holder [Salmonella phage Vi01]; PF00004.22 AAA Family |
| orf165 | 93070 - 93738 | 669 | - | 24662 | 4.8 | 222 | gp45 sliding clamp, DNA polymerase accessory protein | CBW38003.1| Gp45-sliding clmap holder [Salmonella phage Vi01]; PF09116.3 gp45-slide_C Domain |
| orf166 | 94079 - 94456 | 378 | + | 13295 | 4.8 | 125 | conserved hypothetical protein | CBW38004.1| putative uncharacterised protein [Salmonella phage Vi01] |
| orf168 | 94447 - 95955 | 1509 | - | 57850 | 6.4 | 502 | RNA-DNA and DNA-DNA helicase UvsW | CBW38005.1| putative type III restriction enzyme [Salmonella phage Vi01]; PF00271.24 Helicase_C & PF04851.8 ResIII Families |
| orf169 | 95984 - 96730 | 747 | - | 28887 | 6.3 | 248 | conserved hypothetical protein | CBW38006.1| conserved uncharacterised protein [Salmonella phage Vi01] |
| orf170 | 96730 - 97182 | 453 | - | 17881 | 5.3 | 150 | putative DNA repair/recombination protein UvsY | CBW38007.1| putative uncharacterised protein [Salmonella phage Vi01]; PF11056.1 UvsY Family |
| orf171 | 97225 - 97725 | 501 | - | 18373 | 4.4 | 166 | gp3 tail completion and sheath stabilizer protein | CBW38008.1| Gp3-tail completion [Salmonella phage Vi01]; PF06841.5 Phage_T4_gp19 Family |
| orf172 | 97755 - 98405 | 651 | + | 24070 | 8.4 | 216 | hypothetical protein | CBW38009.1| putative uncharacterised protein [Salmonella phage Vi01] |
| orf174 | 98407 - 99135 | 729 | - | 25101 | 4.7 | 242 | conserved hypothetical protein | CBW38010.1| phage associated protein [Salmonella phage Vi01] |
| orf175 | 99174 - 99341 | 168 | - | 6642 | 8.8 | 55 | hypothetical protein |  |
| orf176 | 99383 - 99805 | 423 | - | 15909 | 8.5 | 140 | conserved hypothetical protein | CBW38012.1| conserved uncharacterised protein [Salmonella phage Vi01]; PF10686.2 DUF2493 Family |
| orf177 | 99811 - 100131 | 321 | - | 11854 | 10.3 | 106 | hypothetical protein | CBW38013.1| putative uncharacterised protein [Salmonella phage Vi01] |
| orf178 | 100191 - 100472 | 282 | - | 10477 | 5.8 | 93 | hypothetical protein |  |
| orf179 | 100575 - 100811 | 237 | - | 8709 | 6.8 | 78 | conserved hypothetical protein | CBW38015.1| putative uncharacterised protein [Salmonella phage Vi01] |
| orf180 | 100820 - 101260 | 441 | - | 16869 | 5.6 | 146 | hypothetical protein | CBW38017.1| phage associated protein [Salmonella phage Vi01] |
| orf181 | 101273 - 101500 | 228 | - | 8891 | 5.0 | 75 | hypothetical protein |  |
| orf182 | 101561 - 101761 | 201 | - | 7494 | 4.3 | 66 | hypothetical protein | YP_001743234.1| hypothetical protein EcSMS35_1169 [Escherichia coli SMS-3-5] |
| orf183 | 101821 - 102153 | 333 | - | 11974 | 4.7 | 110 | hypothetical protein |  |
| orf184 | 102246 - 102473 | 228 | - | 8614 | 8.0 | 75 | hypothetical protein |  |
| orf185 | 102566 - 103888 | 1323 | - | 47976 | 5.2 | 440 | gp23, major head protein | CBW38020.1| Gp23-Major capsid protein [Salmonella phage Vi01]; PF07068.4 Gp23 Family |
| orf186 | 103980 - 104846 | 867 | - | 31615 | 4.6 | 288 | gp22, prohead core protein | ADQ55857.1| prohead core protein [Escherichia phage PhaxI] & CBW38021.1| Gp22 prohead core scaffold protein [Salmonella phage Vi01] |
| orf188 | 104892 - 105560 | 669 | - | 24590 | 5.2 | 222 | gp21, prohead core scaffold and protease | CBW38022.1| Gp21 prohead protease [Salmonella phage Vi01]; PF03420.6 Peptidase_U9 Family |
| orf189 | 105568 - 105873 | 306 | - | 11743 | 11.2 | 101 | conserved hypothetical protein | CBW38023.1| conserved phage assoiated protein [Salmonella phage Vi01] & ABC95188.1| GP68-prohead core protein [Stenotrophomonas phage SMB14] |
| orf190 | 105884 - 106051 | 168 | - | 5771 | 3.8 | 55 | hypothetical protein | CBW38024.1| putative uncharacterised protein [Salmonella phage Vi01] |
| orf191 | 106089 - 107780 | 1692 | - | 63246 | 4.9 | 563 | gp20 portal vertex protein of head | CBW38025.1| Gp20 portal vertex protein of the head [Salmonella phage Vi01]; PF07230.4 Phage_T4_Gp20 Family |
| orf192 | 107847 - 108380 | 534 | - | 19783 | 4.8 | 177 | gp19 tail tube protein | CBW38026.1| Gp19 tail tube protein [Salmonella phage Vi01]; PF06841.5 Phage_T4_gp19 Family |
| orf195 | 108459 - 110354 | 1896 | - | 68216 | 4.8 | 631 | gp18 tail sheath protein | CBW38028.1| Gp18 tail sheath protein [Salmonella phage Vi01]; PF04984.7 Phage_sheath_1 Family |
| orf196 | 110407 - 112617 | 2211 | - | 84567 | 5.3 | 736 | gp17 terminase DNA packaging enzyme, large subunit | CBW38029.1| Gp17 terminase subunit for DNA packaging, nuclease and ATPase [Salmonella phage Vi01]; PF03237.8 Terminase_6 Family |
| orf198 | 112598 - 113299 | 702 | - | 25453 | 4.5 | 233 | gp16 terminase DNA packaging enzyme small subunit | CBW38030.1| Gp16 terminase DNA packaging enzyme small subunit [Salmonella phage Vi01]; PF11053.1 DNA_Packaging Family |
| orf199 | 113302 - 113997 | 696 | - | 26696 | 5.2 | 231 | gp15 proximal tail sheath stabilization protein | CBW38031.1| Gp15 proximal tail sheath stabilization [Salmonella phage Vi01] |
| orf200 | 114000 - 114650 | 651 | - | 24908 | 4.4 | 216 | gp14 neck protein | CBW38032.1| Gp14 neck protein [Salmonella phage Vi01]; PF11649.1 T4_neck-protein Family |
| orf201 | 114710 - 114922 | 213 | + | 7795 | 4.8 | 70 | conserved hypothetical protein | CBW38033.1| putative uncharacterised protein [Salmonella phage Vi01] |
| orf202 | 114950 - 115702 | 753 | - | 28724 | 4.9 | 250 | gp13 neck protein | CBW38034.1| Gp13 neck protein [Salmonella phage Vi01] |
| orf203 | 115692 - 116030 | 339 | - | 12225 | 6.2 | 112 | conserved hypothetical membrane protein | CBW38035.1| putative uncharacterised protein [Salmonella phage Vi01]; one TMD |
| orf204 | 116014 - 116262 | 249 | - | 8581 | 5.5 | 82 | conserved hypothetical protein | CBW38036.1| putative uncharacterised protein [Salmonella phage Vi01] |
| orf206 | 116313 - 121151 | 4839 | - | 177204 | 4.9 | 1612 | conserved hypothetical protein | CBW38037.1| conserved phage associated protein [Salmonella phage Vi01] |
| orf207 | 121250 - 123034 | 1785 | - | 63377 | 8.0 | 594 | conserved hypothetical protein | CBW38038.1| tail spike protein [Salmonella phage Vi01] & CAO78738.1| tailspike protein [Salmonella phage Det7] |
| orf210 | 123236 - 125497 | 2262 | - | 80040 | 4.7 | 753 | tailspike protein | CBW38039.1| maturation/adhesion protein [Salmonella phage Vi01] |
| orf212 | 125698 - 127719 | 2022 | - | 72819 | 5.9 | 673 | tailspike protein | ADE87922.1| conserved phage protein [Enterobacteria phage Eco1230-10] |
| orf213 | 127766 - 130825 | 3060 | - | 106911 | 4.7 | 1019 | conserved hypothetical protein | CBW38041.1| hemolysin-type calcium-binding protein [Salmonella phage Vi01] & YP_002003830.1| gp17 [Klebsiella phage K11] |
| orf214 | 130878 - 132089 | 1212 | - | 43037 | 4.8 | 403 | conserved hypothetical protein | CBW38042.1| putative uncharacterised protein [Salmonella phage Vi01] |
| orf215 | 132092 - 132946 | 855 | - | 33369 | 4.7 | 284 | conserved hypothetical protein | CBW38043.1| phage associated protein [Salmonella phage Vi01] |
| orf217 | 132927 - 134708 | 1782 | - | 65062 | 4.6 | 593 | gp6 baseplate wedge subunit | CBW38044.1| Gp6 baseplate wedge subunit [Salmonella phage Vi01] & YP_003358919.1| gp6 baseplate wedge subunit [Deftia phage phiW-14]; PF04865.7 Baseplate_J Family |
| orf218 | 135058 - 135630 | 573 | + | 21648 | 9.2 | 190 | conserved hypothetical protein | CBW38045.1| putative uncharacterised protein [Salmonella phage Vi01] |
| orf219 | 135688 - 135834 | 147 | + | 5136 | 4.2 | 48 | conserved hypothetical protein | CBW38046.1| putative uncharacterised protein [Salmonella phage Vi01] |
| orf220 | 137998 - 138195 | 198 | + | 7313 | 5.5 | 65 | conserved hypothetical protein | NP_944054.1| hypothetical protein Aeh1p176 [Aeromonas phage Aeh1] |
| orf223 | 139058 - 139234 | 177 | + | 6754 | 9.7 | 58 | hypothetical protein |  |
| orf224 | 139577 - 140230 | 654 | + | 24885 | 9.4 | 217 | conserved hypothetical protein | CBW38049.1| putative uncharacterised protein [Salmonella phage Vi01] |
| orf225 | 140287 - 140796 | 510 | + | 18845 | 8.9 | 169 | hypothetical protein |  |
| orf226 | 140879 - 142135 | 1257 | + | 47391 | 7.1 | 418 | hypothetical protein | CBW38050.1| putative uncharacterised protein [Salmonella phage Vi01] |
| orf227 | 142281 - 142739 | 459 | + | 16676 | 3.3 | 152 | conserved hypothetical protein | CBW38052.1| phage associated protein [Salmonella phage Vi01] |
| orf229 | 142767 - 143138 | 372 | - | 14362 | 6.4 | 123 | hypothetical protein |  |
| orf230 | 143135 - 143521 | 387 | - | 14845 | 9.2 | 128 | hypothetical protein |  |
| orf232 | 143570 - 144127 | 558 | - | 20590 | 4.2 | 185 | conserved hypothetical protein | CBW38055.1| putative uncharacterised protein [Salmonella phage Vi01] |
| orf233 | 144223 - 144435 | 213 | - | 7729 | 10.0 | 70 | conserved hypothetical protein | CBW38056.1| putative uncharacterised protein [Salmonella phage Vi01] |
| orf234 | 144478 - 145050 | 573 | - | 20583 | 6.8 | 190 | conserved hypothetical protein | CBW38057.1| putative uncharacterised protein [Salmonella phage Vi01] |
| orf236 | 145130 - 148129 | 3000 | + | 116506 | 5.8 | 999 | gp43 DNA polymerase | CBW38058.1| Gp43 DNA polymerase [Salmonella phage Vi01]; PF00136.14 DNA_pol_B & PF03104.12 DNA_pol_B_exo Families |
| orf237 | 148192 - 148533 | 342 | + | 12323 | 4.2 | 113 | conserved hypothetical protein | CBW38059.1| putative uncharacterised protein [Salmonella phage Vi01] |
| orf239 | 148530 - 149309 | 780 | + | 30044 | 5.3 | 259 | conserved hypothetical protein | CBW38060.1| putative uncharacterised protein [Salmonella phage Vi01]; PF06941.5 NT5C Family |
| orf240 | 149319 - 149624 | 306 | + | 11549 | 4.6 | 101 | conserved hypothetical protein | CBW38061.1| putative uncharacterised protein [Salmonella phage Vi01] |
| orf242 | 149624 - 150310 | 687 | + | 25543 | 9.6 | 228 | segD homolog (homing endonuclease) | NP_899393.1| SegD [Vibrio phage KVP40]; PF01541.17 GIY-YIG Domain |
| orf243 | 150303 - 151220 | 918 | + | 34305 | 8.5 | 305 | conserved hypothetical protein | CBW38062.1| putative uncharacterised protein [Salmonella phage Vi01] |
| orf244 | 151223 - 151420 | 198 | + | 7037 | 9.3 | 65 | hypothetical membrane protein | two TMB |
| orf245 | 151417 - 151797 | 381 | + | 14166 | 5.6 | 126 | conserved hypothetical protein | CBW38064.1| putative uncharacterised protein [Salmonella phage Vi01] |
| orf246 | 151778 - 151963 | 186 | + | 7024 | 9.6 | 61 | hypothetical membrane protein | two TMD |
| orf247 | 151960 - 153186 | 1227 | + | 48369 | 5.7 | 408 | conserved hypothetical protein | CBW38065.1| putative uncharacterised protein [Salmonella phage Vi01] |
| orf248 | 153223 - 153390 | 168 | + | 6077 | 6.5 | 55 | conserved hypothetical membrane protein | CBW38066.1| putative uncharacterised protein [Salmonella phage Vi01]; one TMD |
| orf249 | 153473 - 153685 | 213 | + | 7857 | 5.3 | 70 | hypothetical protein |  |
| orf250 | 153682 - 154068 | 387 | + | 14870 | 6.7 | 128 | hypothetical protein | CBW38068.1| putative uncharacterised protein [Salmonella phage Vi01] |
| orf251 | 154065 - 155177 | 1113 | + | 42047 | 8.2 | 370 | conserved hypothetical membrane protein | CBW38069.1| conserved uncharacterised protein [Salmonella phage Vi01]; three TMD |
| orf252 | 155196 - 155765 | 570 | + | 21218 | 7.6 | 189 | conserved hypothetical membrane protein | CBW38070.1| conserved uncharacterised protein [Salmonella phage Vi01]; one TMD |
| orf253 | 155770 - 155898 | 129 | + | 4840 | 4.9 | 42 | hypothetical protein |  |
| orf255 | 155898 - 156323 | 426 | + | 15546 | 5.6 | 141 | conserved hypothetical protein | CBW38071.1| putative uncharacterised protein [Salmonella phage Vi01] |
| orf256 | 156386 - 156748 | 363 | + | 13871 | 10.0 | 120 | conserved hypothetical protein | CBW38072.1| putative uncharacterised protein [Salmonella phage Vi01] |
| orf257 | 156748 - 157200 | 453 | + | 16607 | 4.3 | 150 | conserved hypothetical protein | CBW38073.1| putative uncharacterised protein [Salmonella phage Vi01] |
| orf258 | 157197 - 157397 | 201 | + | 7194 | 11.1 | 66 | hypothetical protein |  |
| orf259 | 157369 - 157542 | 174 | + | 6556 | 8.1 | 57 | conserved hypothetical protein | CBW38075.1| putative uncharacterised protein [Salmonella phage Vi01] |
| orf260 | 157553 - 157900 | 348 | + | 12953 | 4.8 | 115 | conserved hypothetical protein | CBW38076.1| putative uncharacterised protein [Salmonella phage Vi01] |

BLASTP and PFAM searches conducted on January 12, 2011. The minimum E value used for reporting the PFAM values was 8.4 e-05. TMD = transmembrane domains
