# Supplementary material for: A Shigella boydii bacteriophage which resembles Salmonella phage ViI
Source: Virol J. 2011 May 19;8:242. doi: 10.1186/1743-422X-8-242 (PMC3121705; doi:10.1186/1743-422X-8-242)
Supplement: Additional file 2 — Table S2. Putative promoter and rho-independent terminators found in the ΦSboM-AG3 genome. [file 1743-422X-8-242-S2.DOC]

Additional Table S2. Putative promoter and rho-independent terminators found in the AG3 genome.

A. Early promoters:

Name Coordinates Strand Sequence

P_orf009_ 6563..6590 + TTttCAtttccagcattcggtgTATAAT

P_orf067_ 38622..38648 - TTcAatagatgaaggggcTGcTATAAT

P_orf069_ 39603..39630 - TTtACAtttatgaaaaatgcagTATAtT

P_orf100_ 58420..58446 - TTGAaaaagactttactcttcaATAAT

P_orf138_ 79794..79821 - TTttacttcttcaataactcacgTATAAT

P_orf170_ 97212..97241 - TTGAttctaatccatgaaatacggTATAAT

P_orf206_ 121176..121203 - TTGgCtgtatactaaatatctcTATAAT

P_tRNA-Ser_ 137657..137686 + TTGACAgtccttggttggtcacacaATAAT

P_orf237_ 148126..148155 + TTGAttcaataaaccaaagggggaTATAAT

Nucleotides found in consensus TTGACA(N15-17)TATAAT in capital letters.

B. Putative late promoters

P_orf072_ 41524..41552 tctttgccatttgtcaactcctataaata

P_orf084_ 47655..47683 tataaggcaaaatccgctgcgcataaata

P_orf217_ 134939..134967 tgtagaaaagtagtttttgcctattaata


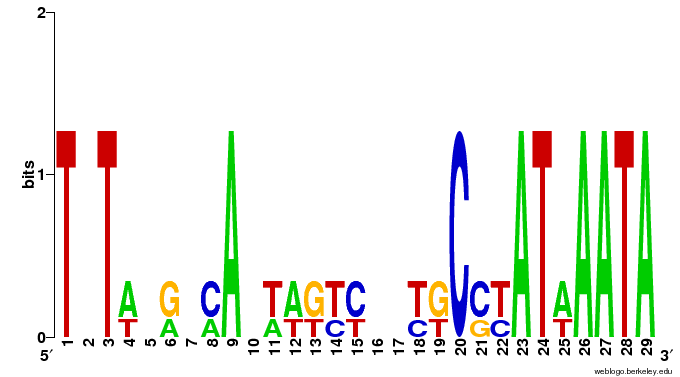


C. Rho-independent terminators

Name Coordinates Strand Sequence Stability (ΔG)

T_orf002_ 4370..4393 + gggggcttcggcctccttttcttt -14.00 kcal/mol

T_orf009_ 7156..7176 + ggggaggaaactcccctattt -15.40

T_orf021_ 12894..13304 + ggcggtatcattaccgcctt -12.60

T_orf027_ 15120..15137 + gccccgtgaggggctttt -14.10

T_orf031_ 16904..16933 + gggggagtatatacttccccttagtttatt -13.10

T_orf036_ 18421..18445 + cccgctccgttggggcgggtttaat -14.20

T_orf049_ 28630..28655 - gaggggcgaaagcccctcattcattt -19.10

T_orf057_ 31631..31659 - gcccagtcaagtactgggctttttaattt -12.00

T_orf069_  38602..38635 - ggggctgctataatagcagctccttttgtttatt -16.30

T_orf073_ 42535..42555 + gccctccgaagagggcttgtt -11.10

T_orf074_ 42526..42550 - gccctccgaagagggcttgtt -11.10

T_orf075_ 43688..43927 - cggggtataattaccccgctttgt -11.70

T_orf083_ 47356..47380 + atcccgcttcggcgggatttttatt -14.70

T_orf088_ 50666..50691 - gcccctcattgaggggctttactttt -13.30

T_orf093_ 52346..52371 - ggggtgagatatcaccccttattcat -13.30

T_orf095_ 54713..54749 - cgccgccttcaatctacgagggcggcgtataataaaa -19.50

T_orf098_ 57235..57266 - ccttcgcattcattgcggaggtattttcttat -12.20

T_orf100_ 57814..57838 - gggggaggaaactcccctccttatt -15.90

T_orf101_ 58472..58499 - ggcctcgtcgaatgaggccttttaaaat -12.30

T_orf105_ 60826..60856 - tccccctaaatacagggggatttctttttct -12.70

T_orf127_ 74388..74413 - gccggagcattgtactccggcttttt -13.30

T_orf131_ 76060..76092 - gggcgacttatcataaggtcgcccttttcattt -16.00

T_orf138_ 79462..79488 - ggcgaggtatgattgcctcgcccattt -16.70

T_orf139_ 79759..79783 - gggcgggaatagttcccgcctgttt -15.10

T_orf141_ 80191..80218 - gccccaacatttgttggggctttacttt -15.90

T_orf144_ 80563..80586 - gggcggggcaacccgcctttcttt -16.70

T_orf149_ 83331..83352 - ggggaggcaactcccctgtttt -14.90

T_orf152_ 85053..85085 - gacgcccagtatatttgctgggtgtttcaattt -16.80

T_orf155_ 86303..86327 - ccccgccatgtgcggggtttctttt -11.50

T_orf160_ 88811..88837 - gcgccctggcaaacggggcgcacattt -17.80

T_orf165_ 93016..93040 - gggcgggggcgttccccgcctattt -18.80

T_orf185_ 102516..102542 - ccccgcttcggcggggtttttcattat -15.50

T_orf201_ 114928..114953 + gggcgggggaacccgcctttccttat -15.70

T_orf202_ 114921..114944 - ggcgggttcccccgcccttctttt -11.90

T_orf207_ 121210..121241 - gcccctcagatgaggggctttttctttctatt -15.20

T_orf210_ 123207..123229 - ggtcccgaaagggaccttttctt -15.30

T_orf223_ 139058..139234 + gccaggtaaggaaacctggctttctttt -12.70

T_orf227_ 142746..142768 + ggggcattaagcccctttctttt -10.40

T_orf229_ 142732..142760 - ggggcttaatgcccctttttgttattctt -10.9

T_orf248_ 153419..153457 + ggggaatgggttaatattagcccgttcccctttcttttt -22.90

Nucleotides in stem structures underlined
