# Supplementary material for: A Shigella boydii bacteriophage which resembles Salmonella phage ViI
Source: Virol J. 2011 May 19;8:242. doi: 10.1186/1743-422X-8-242 (PMC3121705; doi:10.1186/1743-422X-8-242)
Supplement: Additional file 3 — Table S3. Details of mass spectrometric analysis of ΦSboM-AG3 proteins. [file 1743-422X-8-242-S3.DOC]

**Additional Table S3.** Details of mass spectrometric analysis of ФSboM-AG3 proteins

| **m/z**  **(Meas.)** | **[MH+]**  **(calc.)** | **error**  **(Da)** | **Peptide** | **Sequence** |
| --- | --- | --- | --- | --- |
| Band A | gene_orf00206; |conserved hypothetical phage protein ,  MW: 177203.11; Sequence coverage: 39.6% | | | |
| 0001 MQEMNLNRR**P YWDDWNPEK**R FSR**ILFRPAP IK**VQTR**ELNQ MQTIFQDQLE K**LGNHLFKDG SMVIPGGLTI  0071 TNTAVSMK**FT LAGGSEFTDL EGISELYVLG K**DNNAKARVL SLERYLAEPD TMYAILEMTE SGSSDGFEAG  0141 DNLYFNTYDV NDNFIRVGYG VAATVGGSIV ARMTK**GVYFV RGMFLDVEAA TLIVDNASNS TSHR**VGFK**VT**  0211 **ETIVTETEDE SLFSNAQGTP NSKAPGAHRL RIDLVLSRYG YDEEVADFVE LAK**VKDGR**IQ SMVTQSTYNI**  0281 **LEDSMAQRTY ETNGDYNVST HQIDLREHLK** ENNNGGVYTP AEGGDASK**FV AVMKPGISYV R**GR**RIENLGE**  0351 **ELVTIDKARD TDTLNNNPVA VATGNYLVSK** NSKGVPVVSR TIRYK**FLNAS SAVQATALLI SAER**SGAEFR  0421 LYMRDLVVTG DMGTVTKVAY EESGTTMFSC ELESNQFSQS SAIDLIFPLP VFGVKTLAPT GTIDINYTVL  0491 RSNK**ITLNAA GAGSISAPLG YSFSPEFSLY SAAK**ADGTAA QFDISSGLSL TGTPVGSALQ ISLGAGYANQ  0561 SINLLALMVR TTATIKTK**TV TEVTETVTFT SAASVQLNNH DGYK**LVSVK**N ASGADVTSNF TLDGGQR**DAA  0631 YYRSNLLSGT GAISGTFTVV YQYFAHSSGD FFTADSYSSI DYPDIPNYVS SSSGTVYGLA DCLDFRPK**IT**  0701 **SGASDTDMVR PNTAIVLDAE YYLPRIDAVY LADNGVFNVA R**GVSSNNLAS PAIPDNGMR**L YELMIPPYTA**  0771 **NIDDIQIR**TI DNRRYTMR**DI GKLETRISNV EYYTSLSQLE SSAMTQQVFD PITGNPR**FKN GIAADPFKDF  0841 R**LIDDLSADW VGSIDTDNGR** **LRPFVQQNAV DLTPVGWNNV QDGMVVCNYT REISVRQDYA TTTINVNPYA**  0911 **VFNWEGFLKL NPTTDYWFEN YYVAPR**VINE TINTRGAIKE GSVYGTWRTV SVSER**VWEPH GAGGVWWGYR**  0981 **YR**TTVSTRDV TTYTYTDK**TT TTLTGEQIVE TQVIPYMRKT NINFEATGLR PFTRVYPFFS GR**DVSAYCTP  1051 NGGSLGGALN TDANGNIK**GV FAVPQNETVK FNTGDNVFRL TDSPVDSKSA DDTLTNAEAV HK**SFGKKQGI  1121 QK**TYVNTRVL GYTATK**STEN KTETVEVAKW KDPIAQSFMV ATNIGGEYIE GIEVFFSTKS RDIPITLEIR  1191 EMSNGLPATT VVTRKTLNPS EVSVSTDSSV PTMFKFDYPV YLQAQTEFAI VLLANTQDYN AYIAEMGKKN  1261 NLLTNEYIAK QPYTGVFFTS SNGTTWTPNQ TDMKFRVYRC NFSAGSNIVT FDAKVGPKIR PLGLNTVKCV  1331 NGSSTVTVYA PGHGLTAGES VTLSGLTGGC GFTPEQLNAQ HTVTDATFTT FKFVLSSNAD SDGQIGGEEA  1401 MSFLGNYLVD MFYASVTNSA LEGSVLKLEY RYRDATSNSF SDWAEFESDT DVSLSTEGIY RQVGDFQVRA  1471 TTRNESNVYT APMIDGDDFT IIFNTYGVDP FEDVFSYVTK DIGFDNPCTT LKQYFGAMLP SQSSMTVQVK  1541 LLRAGQEMDD VAWETVTPTS PLVNDGSTFF EYEYDKTVDS TNPFVGLKIR LLVRGNRTAP PSFKDFRLIA  1611 LA | | | | |
| 972.494 | 972.494 | 0.000 | 1035-1042 | VYPFFSGR |
| 1054.674 | 1054.677 | -0.003 | 24-32 | ILFRPAPIK |
| 1069.509 | 1069.506 | 0.003 | 1081-1089 | FNTGDNVFR |
| 1288.687 | 1288.690 | -0.003 | 1069-1080 | GVFAVPQNETVK |
| 1471.704 | 1471.702 | 0.002 | 1099-1112 | SADDTLTNAEAVHK |
| 1482.819 | 1482.813 | 0.006 | 329-341 | FVAVMKPGISYVR (Met-OX) |
| 1505.689 | 1505.681 | 0.008 | 9-19 | RPYWDDWNPEK |
| 1586.846 | 1586.854 | -0.008 | 1123-1136 | TYVNTRVLGYTATK |
| 1628.883 | 1628.885 | -0.002 | 344-357 | RIENLGEELVTIDK |
| 1736.897 | 1736.897 | 0.000 | 726-741 | IDAVYLADNGVFNVAR |
| 1747.808 | 1747.806 | 0.002 | 249-263 | YGYDEEVADFVELAK |
| 1809.837 | 1809.836 | 0.001 | 610-627 | NASGADVTSNFTLDGGQR |
| 1880.907 | 1880.906 | 0.001 | 37-51 | ELNQMQTIFQDQLEK (Met-OX) |
| 1962.055 | 1962.066 | -0.011 | 396-414 | FLNASSAVQATALLISAER |
| 2011.962 | 2011.972 | -0.010 | 1081-1098 | FNTGDNVFRLTDSPVDSK |
| 2061.971 | 2061.972 | -0.001 | 842-860 | LIDDLSADWVGSIDTDNGR |
| 2075.992 | 2075.999 | -0.007 | 966-982 | VWEPHGAGGVWWGYRYR |
| 2148.993 | 2149.003 | -0.010 | 920-936 | LNPTTDYWFENYYVAPR |
| 2206.097 | 2206.099 | -0.002 | 360-380 | DTDTLNNNPVAVATGNYLVSK |
| 2294.155 | 2294.174 | -0.019 | 760-778 | LYELMIPPYTANIDDIQIR (Met-OX) |
| 2347.098 | 2347.090 | 0.008 | 269-288 | IQSMVTQSTYNILEDSMAQR (Met-OX) |
| 2425.256 | 2425.263 | -0.007 | 999-1019 | TTTTLTGEQIVETQVIPYMRK |
| 2433.232 | 2433.237 | -0.005 | 358-380 | ARDTDTLNNNPVAVATGNYLVSK |
| 2446.233 | 2446.239 | -0.007 | 79-101 | FTLAGGSEFTDLEGISELYVLGK |
| 2544.272 | 2544.287 | -0.015 | 242-263 | IDLVLSRYGYDEEVADFVELAK |
| 2633.236 | 2633.259 | -0.013 | 289-310 | TYETNGDYNVSTHQIDLREHLK |
| 2690.376 | 2690.384 | -0.008 | 1020-1042 | TNINFEATGLRPFTRVYPFFSGR |
| 2697.276 | 2697.274 | 0.002 | 209-233 | VTETIVTETEDESLFSNAQGTPNSK |
| 2812.350 | 2812.364 | -0.014 | 579-604 | TVTEVTETVTFTSAASVQLNNHDGYK |
| 2984.467 | 2984.467 | 0.000 | 699-725 | ITSGASDTDMVRPNTAIVLD AEYYLPR (Met-OX) |
| 2990.492 | 2990.515 | -0.023 | 495-524 | ITLNAAGAGSISAPLGYSFSPEFSLYSAAK |
| 3185.559 | 3185.568 | -0.009 | 176-204 | GVYFVRGMFLDVEAATLIVDNASNSTSHR (Met-OX) |
| 3275.631 | 3275.637 | -0.006 | 892-919 | EISVRQDYATTTINVNPYAV FNWEGFLK |
| 3402.759 | 3402.780 | -0.021 | 234-263 | APGAHRLRIDLVLSRYGYDE EVADFVELAK |
| 3491.653 | 3491.664 | -0.011 | 799-827 | ISNVEYYTSLSQLESSAMTQQVFDPITGNPR (Met-OX) |
| 3606.731 | 3606.758 | -0.027 | 861-891 | LRPFVQQNAVDLTPVGWNNV QDGMVVCNYTR |
|  |  |  |  |  |
| Band B | gene_orf00213; conserved hypothetical phage protein  MW: 106909.85 Da; Sequence coverage: 42.6% | | | |
| 001 MANKPTQPVF PLGLVAEEQS TLAGILNTGT IEHGPDAVLT LPEGNASAGL PSSVRYNADS  061 DEFEGFYENG GWLPLGGGGI RWEALPHAST ATLTEGR**GYL VDNSTGVSTV VFPSPTRIGD**  121 **SVTVCDLYGK** FSLYPLTIDP NGHPMYGSVE PMTLSTDSVS ATFTWSGDAR GWIVTAGVGL  181 GQGRVYSR**TI FTETVASDTA QVTLTTQPSI VDVYVDGKR**L LESKYSLNGF NVDFSPSIPS  241 GSELQVIQYV PIQLGDGSGG SGGGTVITWI YNSGSAVGGE TEIELDVDAE DVSEIFIDGS  301 RQQK**GLGFTY DSVTKIITLA DELEAGDEVV VVINGDPTLY NQIDRTPNEV ARSVNVPNSQ**  361 **VILSSDTITK LDGKTVIYDV VAQKIWGLPS GIPTGASIVS VSGSNLSYAP GNVVVPLLPA**  421 **PGSK**DALEAY KGELLAGNTG LVGANAVVVT PQGTLAEMQY YVTPEQFSHL VTAGEYVDEN  481 TDFTLAVQGA VDYAASHPGV IVRGTEK**VYG VGRVLVTVGV K**VIDGLKLK**C IVANTDTLLY**  541 **SFVDTGHTDL QIR**NCILNGN NNTRK**GIIVS GVIRATIEKN YVYGLDGTGE AYGIRIGTTS**  601 **TTSMNINNK**I SENVIEMPTD PWAGTGNYAI CGIGMIGQIT SLYGGLDTNA GVPLFPSTIT  661 LR**DTIIEGNF ISGGTHGVQG LGLFRTLITK** **NHIIGNTHRN INLSPNCQR**V NVVGNLLIDG  721 GSSGVNVAWG CR**WINISGNH IQTSTAAVSP SDDAAIQLYK** GVDQCTVSGN TILGDWKYSV  781 YMGAGVTNVS VNANGLFAGS LASIAVESDW VLTADYPLAI YSSSR**NPNTT PIAGDTGNIN**  841 **IGGNAYGAGS CAIYLAATNN K**AMYNVNIHD EVINSVTSRP HVVYAYDAGT LMTDGSLTNI  901 AARGATTSKY YLSR**GRGAFN VIRDVTALDD PKGEVTVSGG TPSAVFGPNL YIASGTITDF**  961 **TGAQSGDIIN LR**MGDGVVLT HNSTVMRLKG GVNATASGGL AIMTLQRRAG IWFEMSRNF | | | | |
| 776.434 | 776.441 | -0.007 | 917-923 | GAFNVIR |
| 989.553 | 989.564 | -0.009 | 915-923 | GRGAFNVIR |
| 1061.551 | 1061.560 | -0.009 | 691-699 | NHIIGNTHR |
| 1135.635 | 1135.636 | -0.001 | 375-384 | TVIYDVVAQK |
| 1187.588 | 1187.594 | -0.006 | 305-315 | GLGFTYDSVTK |
| 1215.582 | 1215.590 | -0.008 | 700-709 | NINLSPNCQR |
| 1426.683 | 1426.688 | -0.005 | 118-130 | IGDSVTVCDLYGK |
| 1445.882 | 1445.884 | -0.002 | 508-521 | VYGVGRVLVTVGVK |
| 1455.884 | 1455.889 | -0.005 | 566-579 | GIIVSGVIRATIEK |
| 1747.823 | 1747.828 | -0.005 | 580-595 | NYVYGLDGTGEAYGIR |
| 1902.012 | 1902.018 | -0.006 | 353-370 | SVNVPNSQVILSSDTITK |
| 2096.060 | 2096.066 | -0.006 | 98-117 | GYLVDNSTGVSTVVFPSPTR |
| 2315.229 | 2315.245 | -0.016 | 353-374 | SVNVPNSQVILSSDTITKLDGK |
| 2388.231 | 2388.231 | -0.000 | 663-685 | DTIIEGNFISGGTHGVQGLGLFR |
| 2752.354 | 2752.361 | -0.007 | 530-553 | CIVANTDTLLYSFVDTGHTDLQIR |
| 2944.573 | 2944.589 | -0.016 | 663-690 | DTIIEGNFISGGTHGVQGLG LFRTLITK |
| 3000.501 | 3000.506 | -0.005 | 733-760 | WINISGNHIQTSTAAVSPSDDAAIQLYK |
| 3199.624 | 3199.626 | -0.004 | 189-218 | TIFTETVASDTAQVTLTTQPSIVDVYVDGK |
| 3226.518 | 3226.532 | -0.014 | 580-609 | NYVYGLDGTGEAYGIRIGTTSTTSMNINNK (Met-OX) |
| 3284.701 | 3284.690 | 0.011 | 316-345 | IITLADELEAGDEVVVVINGDPTLYNQIDR |
| 3355.701 | 3355.727 | -0.016 | 189-219 | TIFTETVASDTAQVTLTTQPSIVDVYVDGKR |
| 3565.685 | 3565.698 | -0.013 | 826-861 | NPNTTPIAGDTGNINIGGNAYGAGSCAIYLAATNNK |
| 3858.981 | 3859.100 | -0.019 | 385-424 | IWGLPSGIPTGASIVSVSGSNLSYAPGNVVVPLLPAPGSK |
| 3982.990 | 3983.003 | -0.013 | 933-972 | GEVTVSGGTPSAVFGPNLYIASGTITDFTGAQSGDIINLR |
| 4052.093 | 4052.082 | 0.011 | 316-352 | IITLADELEAGDEVVVVING DPTLYNQIDRTPNEVAR |
| 2944.573 | 2944.589 | -0.016 | 663-690 | DTIIEGNFISGGTHGVQGLG LFRTLITK |
|  |  |  |  |  |
| Band C | gene_orf00195; Gp18 tail sheath protein  MW: 68215.33 Da; Sequence coverage: 43.1% | | | |
| 001 MATQSFSVAP SVQWTERDAT LQTSPSVVVQ GATVGK**FQWG EAELPVLVTG**  051 **GETGLVKK**FF KPNDATATDF LVIADFLSYS SVAWVTRVVG PAARNAVTKG  101 QTAILIRNKL DFETASPSAS ITWTGRYAGS LGNDVAINVC DAAGFPTWEF  151 R**NNFAYAPQA GEYHIVIVDK** VGRITDSSGA VGQVDR**ISVS GTATGAGSIS**  201 **VAGEDVAYTD TDTPATLATK** **IGTALTALTD VYSSVVVK**SN TVTVTHK**AIG**  251 **PQTVTAIVPD ANGLTATAVT TTVGASGSII EK**YELMQATQ GSKKSDGSNA  301 YFKDVINDTS NWVYTFATTL AAGVTELEGG VDDYTGNR**VA AIEALNNAEA**  351 **YDAKPVFAFC EELIEQQTLI DLSTERKDTV SFVSPLRDVV VGNR**GREMED  401 VVAWRESLVR DSSYFFMDDN WAYVYDKYND KMRWIPACGG TAGVWAR**SIE**  451 **IAGIYK**SPAF HNRGKYNNYN RMAWSASSDE R**AVLYRNQIN SIVTFSNEGI**  501 **VLYGDKTGLT RPSAFDRINV RGLFIMAEQN IAAIAKYYLG ENNDEFTRSL**  551 **FSNAVRPYIR** QLANMGAIYD GQVKCDADNN TADIIAANQM VAGIWLKPEY  601 SINWVYLDFA AVRPDMEFSE IETGGGIVAA S | | | | |
| 993.561 | 993.562 | -0.002 | 448-456 | SIEIAGIYK |
| 1120.597 | 1120.600 | -0.003 | 378-387 | DTVSFVSPLR |
| 1220.636 | 1220.638 | -0.002 | 507-517 | TGLTRPSAFDR |
| 1422.777 | 1422.785 | -0.008 | 549-560 | SLFSNAVRPYIR |
| 1520.660 | 1520.665 | -0.005 | 537-548 | YYLGENNDEFTR |
| 1605.864 | 1605.867 | -0.003 | 522-536 | GLFIMAEQNIAAIAK (Met-OX) |
| 1702.927 | 1702.935 | -0.008 | 507-521 | TGLTRPSAFDRINVR |
| 1837.024 | 1837.032 | -0.008 | 221-238 | IGTALTALTDVYSSVVVK |
| 1859.990 | 1859.997 | -0.007 | 378-394 | DTVSFVSPLRDVVVGNR |
| 2149.064 | 2149.071 | -0.011 | 152-170 | NNFAYAPQAGEYHIVIVDK |
| 2211.125 | 2211.129 | -0.004 | 487-506 | NQINSIVTFSNEGIVLYGDK |
| 2230.164 | 2230.175 | -0.011 | 37-57 | FQWGEAELPVLVTGGETGLVK |
| 2358.259 | 2358.270 | -0.011 | 37-58 | FQWGEAELPVLVTGGETGLVKK |
| 2813.471 | 2813.483 | -0.012 | 482-506 | AVLYRNQINSIVTFSNEGIV LYGDK |
| 2924.420 | 2924.432 | -0.012 | 537-560 | YYLGENNDEFTRSLFSNAVRPYIR |
| 3227.561 | 3227.580 | -0.019 | 187-220 | ISVSGTATGAGSISVAGEDV AYTDTDTPATLATK |
| 3324.775 | 3324.790 | -0.015 | 248-282 | AIGPQTVTAIVPDANGLTATAVTTTVGASGSIIEK |
| 4410.238 | 4410.217 | 0.021 | 339-377 | VAAIEALNNAEAYDAKPVFA FCEELIEQQTLIDLSTERK |
|  |  |  |  |  |
| Band D | gene_orf00185; Gp23, major head protein  MW: 47974.42Da; Sequence coverage: 64.1% | | | |
| 001 MAKKLVTEQM REQWLPVLQK ESESIQPLSA ENVAVRLLQN QAEWNAKNLG  051 ESDAPGSVNN SVGK**WQPVLI DMAKRLAPIN IAMDFFGVQP LSGPDGQIFA**  101 **LR**AR**QGVGDS SNTQQSRKEL FMQEADSGYS GDGTVQAGDP SGFTQAEIEG**  151 **SGAGVTTIGK** **GMPTTDAELL GTTTNPWARV GITVQK**ATVT AK**SRGLYADY**  201 **SHELR**QDMMA IHGEDVDSIL SDVMVTEIQA EMNR**EFIRTM NFSAVR**FKK**F**  251 **GANGVVDIST DISGR**WALEK WK**YMTFMLEV EANGIGVDTR** RGKGNRVLCS  301 PNVASALAMS GMLDYAPALQ ENTK**LAIDPT GQTFAGVLSN GMRVYIDPYA**  351 **VAEYITLAYK** **GATALDAGIF FAPYVPLEMY RTQGETTFSP R**MAFKTR**YGI**  401 **CANPFVQIPA NQDPQVYVTA DGIAQDSNPY FRK**GLIKGLF | | | | |
| 925.548 | 925.546 | -0.008 | 239-246 | TMNFSAVR |
| 1123.538 | 1123.539 | -0.001 | 382-391 | TQGETTFSPR |
| 1200.644 | 1200.645 | -0.001 | 65-74 | WQPVLIDMAK |
| 1216.629 | 1216.639 | -0.010 | 65-74 | WQPVLIDMAK (Met-OX) |
| 1323.628 | 1323.633 | -0.005 | 195-205 | GLYADYSHELR |
| 1486.743 | 1486.747 | -0.004 | 235-246 | EFIRTMNFSAVR (Met-OX) |
| 1491.706 | 1491.714 | -0.008 | 105-118 | QGVGDSSNTQQSRK |
| 1566.764 | 1566.766 | -0.002 | 193-205 | SRGLYADYSHELR |
| 1607.801 | 1607.802 | -0.001 | 250-265 | FGANGVVDISTDISGR |
| 1735.884 | 1735.897 | -0.013 | 249-265 | KFGANGVVDISTDISGR |
| 1963.982 | 1963.991 | -0.009 | 325-343 | LAIDPTGQTFAGVLSNGMR |
| 1992.030 | 1992.036 | -0.006 | 344-360 | VYIDPYAVAEYITLAYK |
| 2047.975 | 2047.975 | 0.000 | 161-179 | GMPTTDAELLGTTTNPWAR (Met-OX) |
| 2077.951 | 2077.957 | -0.006 | 273-290 | YMTFMLEVEANGIGVDTR |
| 2318.154 | 2318.152 | 0.002 | 361-381 | GATALDAGIFFAPYVPLEMYR |
| 2773.406 | 2773.418 | -0.012 | 161-186 | GMPTTDAELLGTTTNPWARVGITVQK |
| 2903.503 | 2903.512 | -0.009 | 76-102 | LAPINIAMDFFGVQPLSGPDGQIFALR |
| 3043.595 | 3043.618 | -0.023 | 75-102 | RLAPINIAMDFFGVQPLSGPDGQIFALR |
| 3059.606 | 3059.613 | -0.007 | 75-102 | RLAPINIAMDFFGVQPLSGPDGQIFALR (Met-OX) |
| 3422.667 | 3422.672 | -0.005 | 361-391 | GATALDAGIFFAPYVPLEMY RTQGETTFSPR (Met-OX) |
| 3936.994 | 3937.009 | -0.015 | 325-360 | LAIDPTGQTFAGVLSNGMRVYIDPYAVAEYITLAYK |
| 4056.936 | 4056.954 | -0.018 | 398-433 | YGICANPFVQIPANQDPQVYVTADGIAQDSNPYFRK |
| 4180.866 | 4180.877 | -0.011 | 119-160 | ELFMQEADSGYSGDGTVQAGDPSGFTQAEIEGSGAGVTTI GK (Met-OX) |
|  |  |  |  |  |
| Band E | gene_orf00006; putative tail protein  MW: 27617.86Da; Sequence coverage: 16.4% | | | |
| 001 M**PTITVLVAP EVVRNKPETE RLHTVTGTAK** GWEK**TSLNQD PDEILTECK**G  051 LDALLTKSNL QADGVTKVDP TKPIGFEISY EIHDPSAVLT TGLTITPATA  101 GGEVGQVVEL LATVAPANAT YQGVNWYSGD ITKAVHIGGG KFKLLAPGSV  151 TVYGVTIEGG HTDSTVITVA GALALSTDLA ASQDVTAGAD ATFTIAATGG  201 TTPYTYAWYF SDVPGGEGVV IDAGANATAA TASLVITAVD AADEGEYWCV  251 VEDADGHSVT STRCEMAVV | | | | |
| 1393.838 | 1393.841 | -0.003 | 2-14 | PTITVLVAPEVVR |
| 1762.812 | 1762.816 | -0.004 | 35-49 | TSLNQDPDEILTECK |
| 1776.829 | 1776.832 | -0.003 | 34-48 | TSLNQDPDEILTECK (Cys-acrylamide) |
| 1781.943 | 1781.950 | -0.007 | 15-30 | NKPETERLHTVTGTAK |
| 2248.267 | 2248.266 | 0.001 | 2-21 | PTITVLVAPEVVRNKPETER |
| 3156.769 | 3156.774 | -0.005 | 2-30 | PTITVLVAPEVVRNKPETER LHTVTGTAK |
|  |  |  |  |  |
| Band F | gene_orf00192; Gp19 tail tube protein  MW: 19782.17 Da; Sequence coverage: 55.9% | | | |
| 001 M**ATVNEFRAA MSR**GGGVQRQ HR**WRVTVNFP AFVAGADTIR DVSLLAVTTN**  051 **TPTGQLGEIL VPWGGRELPF PGDRR**FEALP ITFINVVNNS AYNAFEVWQQ  101 CINGSNSNR**A AANPDDYFRD VIMELLDAND NVTK**TWTLQG GWPQNLGQLE  151 LDMSAMDSYT QFTVDLR**YFQ AISDK**SL | | | | |
| 836.419 | 836.426 | -0.007 | 2-8 | ATVNEFR |
| 930.466 | 930.468 | -0.002 | 67-74 | ELPFPGDR |
| 971.486 | 971.483 | 0.003 | 168-175 | YFQAISDK |
| 1086.560 | 1086.569 | -0.009 | 67-75 | ELPFPGDRR |
| 1139.516 | 1139.512 | 0.004 | 110-119 | AAANPDDYFR |
| 1368.669 | 1368.669 | 0.000 | 2-13 | ATVNEFRAAMSR (Met-OX) |
| 1677.892 | 1677.896 | -0.004 | 25-40 | VTVNFPAFVAGADTIR |
| 2020.077 | 2020.076 | 0.001 | 23-40 | WRVTVNFPAFVAGADTIR |
| 2694.446 | 2694.446 | 0.000 | 41-66 | DVSLLAVTTNTPTGQLGEILVPWGGR |
| 2810.330 | 2810.330 | 0.000 | 110-134 | AAANPDDYFRDVIMELLDANDNVTK |
| 2826.321 | 2826.325 | -0.004 | 110-134 | AAANPDDYFRDVIMELLDANDNVTK (Met-OX) |
| 4353.316 | 4353.324 | -0.008 | 25-66 | VTVNFPAFVAGADTIRDVSLLAVTTNTPTGQLGEILVPWGGR |
